# Forecasting nominal exchange rates using a dynamic model averaging framework

**DOI:** 10.1016/j.heliyon.2024.e39112

**Published:** 2024-10-11

**Authors:** Martin Časta

**Affiliations:** a Prague University of Economics and Business, Prague, Czech Republic; b Czech National Bank, Prague, Czech Republic

**Keywords:** Exchange rates, Forecasting, Forecast evaluation

## Abstract

This paper presents a dynamic model averaging approach for forecasting nominal exchange rates. This framework encompasses most of the approaches commonly used in the forecasting literature and also allows us to study parameters and model uncertainty in exchange rate forecasting. We focus on nine major trading currency pairs: AUD/USD, CAD/USD, CHF/USD, EUR/USD, GBP/USD, NOK/USD, NZD/USD, SEK/USD, and JPY/USD, where we use data for approximately the last two decades. We empirically show statistically and economically significant exchange rate predictability in the medium and long run, and we also present some findings on predictability even in the short run. We offer several theoretical explanations for these results on predictability.

## 1 Introduction

Exchange rate forecasting is generally considered highly challenging, and most studies illustrate that consistently beating the random walk benchmark is extremely difficult. This was demonstrated in the early studies of Meese and Rogoff [1], [2] and, until recently, has remained essentially unchanged. On the contrary, this paper presents evidence that there is significant predictability of nominal exchange rates in the medium and long run and even some predictability in the short-term horizon, at least regarding the last two decades considered and the currencies considered. The primary contribution of this study lies in the out-of-sample prediction exercise, wherein we provide forecasts of nominal exchange rates utilizing a dynamic model averaging (DMA) approach. This method outperforms the random walk benchmark, particularly in the medium and long run.

The field of exchange rate forecasting has traditionally centred around achieving optimal point forecasts, driven by the widely acknowledged difficulty of predicting exchange rates. Consistently outperforming the random walk benchmark in forecasting has posed a significant challenge, a fact that until the recent decade has remained largely unchanged since the seminal papers by Meese and Rogoff [1], [2]. Consequently, much of the literature has concentrated on the objective of obtaining the best point forecasts. However, considering the uncertainty surrounding exchange rate forecasts is crucial for economic agents when making decisions. While point forecasts provide valuable information about the most likely outcome, they do not convey the complete picture of the range of possible outcomes and associated risks. By incorporating measures of uncertainty, such as the full probability distribution, economic agents can better assess the potential variability and downside risks associated with different exchange rate scenarios.

We offer several explanations for this result. We present substantive evidence that the nominal exchange rates may be mean-reverting, and the inclusion of other explanatory (fundamental) variables implied by different theories does not significantly change the forecast results, at least in the short run and in the medium-term horizon; their contribution is relatively modest. In the long run, we, however, present some evidence that the inclusion of additional variables significantly improves forecasting results. We illustrate these findings by using a dynamic model averaging framework, which implicitly accounts for most of the possible specifications used in exchange rate forecasting, e.g., the forecast of exchange rate based on a monetary model, the Taylor rule model, uncovered interest rate parity model, and purchasing power parity model of exchange rates.^2^ This framework also allows us to study parameters and model uncertainty in exchange rate forecasting, which is closely related to the so-called ‘scapegoat theory of exchange rates’.

From the practical point of view, most of the studies regarding exchange rate predictions are based on the idea that there exists a cointegrating relation between the exchange rate and other variables, and exchange rates converge back to their fundamental value. On the other hand, we argue that an exchange rate forecast capable of defeating a random walk benchmark is sufficient to use lagged exchange rate values and also the use of a direct forecasting approach, which allows filtering out the non-fundamental noise in the short-run periods. We stress that this finding can be considered novel; although we are not the first to make this claim, the use of the dynamic model averaging approach and the possibility to examine time-variation in parameters and the posterior inclusion probabilities further expands the results recently obtained by [3] regarding the fact that level of the exchange rate appears to have significant forecasting power. The results also indicate that the inclusion of additional fundamental variables can further improve exchange rate predictions.

The rest of this paper is structured as follows: In section 2, we present the existing literature regarding exchange rate forecasting. In sections 3 and 4, we describe the data and the methodology used. Finally, in sections 5 and 6, we present empirical results obtained using a dynamic model averaging approach. In this part, we perform out-of-sample forecasting exercise for the AUD/USD, CAD/USD, EUR/USD, CHF/USD, GBP/USD, NOK/USD, NZD/USD, SEK/USD, and JPY/USD nominal exchange rates and compare them to several other commonly used benchmark models (random walk without drift and benchmark models based on simple OLS regression). The rationale behind the inclusion of these currency pairs follows the study by [3], which also examined a similar time period. These currencies are considered the non-dollar G10 currencies, broadly representing the most heavily and most liquidly traded currencies in the world.

## 2 Literature review

Empirical studies regarding exchange rate development are, in many cases, loosely based on economic theory. Most of the empirical forecasting literature is therefore based on the monetary theory of exchange rates, some version of the Taylor (monetary policy) rule, or the articles are based on some version of the validity of purchasing power parity or uncovered interest rate parity. The body of literature on exchange rate forecasting is extensive. Consequently, this article does not provide an exhaustive summary but instead focuses on a select subset of key contributions. For a more comprehensive review of the literature, refer to, for example, [4].

Exchange rate models grounded in monetary theory [5], [6], [7], [8] have been among the most frequently tested empirically. However, these models typically underperform when compared to other forecasting methods, a result first documented in the seminal works of [1], [2]. More recent research, however, indicates that the predictive power of these models can be somewhat enhanced through alternative approaches, such as panel regression techniques [9], [10], [11], [12], or the application of machine learning and Bayesian methods [13], [14], [15]. It is widely recognized that forecasting performance varies with the time horizon selected, and the literature generally suggests that some degree of predictability emerges over the long term [16], [17], [18], [19], [20], [21], [22], [23].

From a practical point of view, defining money empirically poses significant challenges, and many authors use monetary aggregates without considering the composition of these aggregates. Consequently, this article draws on the rapidly growing body of literature examining the role of credit—the primary counterpart to money—and the mechanics of credit creation. Numerous articles within international economics and finance have integrated the concept of credit into their frameworks. For instance, [24] present a two-country model emphasizing the distinction between net payment flows and financial (gross) flows. [25] adopt a portfolio theory-based approach, while [26] and [27] extend the new Keynesian structural model with a banking sector and endogenous money creation into a two-country model. These studies highlight how exchange rates can be shaped by the credit and activities of the banking sector. [28] then shows that credit to corporations has forecasting power in predicting exchange rates, as it is a key driver in the creation of new deposits (money) connected to real economic activity.

The Taylor (monetary policy) rule inspires other models often used in forecasting literature. This approach is based on the fact that central banks adjust nominal interest rates according to perceived or expected inflation and the output or unemployment gap.^3^ Consequently, this policy rule can be incorporated into the uncovered interest rate parity relation, providing an alternative expression for expected exchange rate movements.^4^ The key (albeit simplified) distinction between this Taylor rule-based approach and the monetary model is that the endogenous monetary policy rate replaces money demand. Several studies have examined this approach and found evidence of forecasting power, including [31], [32], [33], [29], [30], [34], and others. However, the forecasting power is found mostly in the medium and long-run forecasting horizon.

The models based on the validity of purchasing power parity (PPP) are, in theory, the oldest, but from the forecasting point of view, they only recently came back into prominence. [35] show that there exists mean reversion in the real exchange rates due to the long-run convergence towards purchasing power parity, which occurs through nominal exchange rate adjustments. [36] build upon previous results claiming that if real exchange rates are predictable, then the nominal ones are also predictable if one can reasonably extrapolate what drives the relative price indices (differences in the price levels of the given countries). In short, as the authors point out, the building blocks of this approach are that the purchasing power parity holds in the long run and that the nominal exchange rate drives most of the real exchange rate adjustment. In other words, the real exchange rate is mean-reverting.

However, the results of exchange rate predictability are frequently subject to criticism, primarily due to the highly persistent nature of the independent variables [37], [38], [39], [40], [41]. From a theoretical point of view, the situation is even more difficult because if we assume rational expectations, these models generally imply a forward-looking solution. This is a standard asset pricing approach where the exchange rate is a function of discounted values of some (expected) fundamental variables. If the fundamentals (explanatory variables) are highly persistent and the discount factor approaches unity, then the nominal exchange rate behaviour will follow random walk behaviour closely. [42], [43], [11], [44]. Although, this does not negate the fact that these factors may indeed influence exchange rate determination. If the model is correctly specified, the error correction term could serve, in some cases, as an indirect observation of the (mean-reverting) FX risk premium.^5^ For example, [47], [46] claim that the FX risk premium may be a significant determinant and often an omitted variable in forecasting literature.

In terms of the methodology used in this article, we build upon literature that studies parameter and model uncertainty in exchange rate forecasting. Specifically, the studies based on the Bayesian model averaging approach are the most similar ones. These studies often use a more eclectic view regarding theoretical justification and focus more on out-of-sample properties of forecasts. The articles using the BMA/DMA approach are, for example, [48], [49], [50], and [51]. These studies generally show that these approaches outperform more common methods used for exchange rate predictions, primarily as a result of the shrinkage properties of estimators used. Specifically, [14] employ the DMA framework similar to ours for exchange rate forecasting together with a broad array of competing models to examine parameters and model uncertainty. However, unlike us, the authors focus only on short-term predictions. We, on the other hand, explore the predictability in different forecasting horizons and document significant improvements relative to the random walk benchmark only in the medium and long run. More generally, articles by [52], [53], and [54] also examine the role of model uncertainty in exchange rate forecasting.

The parameter and model uncertainty is often theoretically based on the so-called ‘scapegoat theory of exchange rates’,^6^ see, for example, [55], [56], or [57]. This line of reasoning requires a forecasting technique that can handle rapid shifts in parameters and allows the relevant subset of variables to change over time. The DMA framework used in this paper encompasses this theory, as it is able to accommodate both parameter instability and model uncertainty. As emphasized by [52], since the true data-generating process linking fundamentals to the future exchange rate is unknown, a reasonable approach to producing exchange rate forecasts involves searching for an adequate model specification among all possible models believed to be capable of predicting the exchange rate.

In terms of results obtained and the reasoning behind them, the most similar findings are presented in a recent study by [3]. More specifically, the authors present a similar conclusion to our research that the level of nominal exchange rate is the best predictor of future exchange rate changes. However, the authors also present a more skeptical perspective on whether the results obtained may be due to the small sample. In our opinion, this further emphasizes the importance of researching this topic. The use of the dynamic model averaging approach further strengthens the results regarding the forecasting power of the lagged level of exchange rate obtained by [3], at least regarding the short and medium-term horizon. However, our results also show that the DMA approach provides a significantly better forecast in the long run in comparison to the alternative benchmark model, which uses a simple direct multi-step OLS forecast in which the change in the exchange rate is a function of a constant and the lagged level of the real or nominal exchange rate.

## 3 Data

The data used for our forecasting exercise are the spot nominal exchange rates (AUD/USD, CAD/USD, CHF/USD, EUR/USD, GBP/USD, JPY/USD, NOK/USD, NZD/USD, SEK/USD) expressed as USDs per unit of domestic currency and other explanatory (fundamental) variables.^7^ The selection of explanatory variables was primarily guided by the theoretical models explaining exchange rate movements described above and also by empirical studies that were at least partially successful in exchange rate forecasting ([11], [30], [58], and [40]). More specifically, the data used as additional explanatory variables are the countries' price levels, credit, unemployment rates, and interest rates. All variables are transformed to logarithmic form with the exception of interest rates. We use only data in (log) levels; however, adding any data transformation is straightforward in the DMA approach, as is shown later.

Regarding the other explanatory (fundamental) variables used in this study, the rationale for their inclusion is as follows: The motivation behind including interest rates is based on uncovered interest rate parity. The inclusion of unemployment rates is based on monetary policy rule models and, thus, implicitly, on uncovered interest rate parity. The unemployment rate in this regard acts as a proxy for the output/unemployment gap^8^ as in [29] and [30], although we recognize that the time-series properties of these two series are somewhat different. The use of the unemployment rate allows us to avoid various methodological problems associated with measuring the natural rate of unemployment [59]. Additionally, we prefer to use the unemployment rate rather than output (gap) due to the monthly periodicity of the forecasting exercise, as output data is only available quarterly. The inclusion of price levels is motivated by studies by [35], [36] and including these variables, therefore, implicitly accounts for the real exchange rate term in the fundamentals. The inclusion of the proxy variable for money is based on the monetary model of exchange rates. In this case, use credit to non-financial corporations of the given countries denominated in national currencies as a proxy for money, as in [28]. This is due to the fact that this time series is much less persistent than usually used monetary aggregates. Loans to corporations can act as a proxy for corporate deposits, and they should have the largest impact on the nominal exchange rate because they have the highest chance of entering international transactions. More generally, corporate loans are one of the key determinants of real economy dynamics, see [60], and the creation of new deposits has the closest link to the real economy.^9^

Data were obtained from Datastream and a range of national and supranational statistical databases, including those of the Bank of Canada (BoC), Bank of Japan (BoJ), ECB Statistical Data Warehouse, Eurostat, FRED, Reserve Bank of Australia (RBA), Reserve Bank of New Zealand (RBNZ), Statistics Sweden (SCB), and the Swiss National Bank (SNB). We primarily use seasonally unadjusted end-of-period monthly data.^10^ Detailed information about the data can be found in Table A.1 in the Appendix. Table A.2 in the Appendix presents the summary statistics for the final dataset used in the estimation. We should point out that the standard unit root tests mainly indicate the time series' nonstationarity.

We performed our calculations using monthly data from January 1997 to December 2022 in the case of AUD/USD and CAD/USD, in the case of CHF/USD^11^ from January 2002 to December 2022, in case of EUR/USD from January 2003 to December 2022, in the case of GBP/USD from September 1997 to December 2022, in the case of JPY/USD from October 2000 to December 2022, in the case of NZD/USD from June 1998 to December 2022, and in case of SEK/USD from December 2001 to December 2022.

Due to the unavailability of non-revised data for all variables, we relied on the revised dataset available as of December 2022. This choice is unlikely to pose significant issues, as most of the data has not undergone substantial revisions. However, we acknowledge that the unemployment data may have been somewhat affected by revisions. For consistency, we opted not to use earlier versions of the unemployment data, although older vignettes were employed for sensitivity analysis, which yielded results that did not differ significantly. We also abstract from the fact that macroeconomic variables are frequently published with a lag.

The selection of sample periods used in the forecasting exercise was generally made for two reasons. First of all, we were limited by data availability regarding some fundamentals and currencies.^12^ The second reason for the choice of the sample period is to focus on periods dominated by a stable inflation environment and inflation-targeting regimes. However, not including longer time series may somewhat enhance the small sample properties problem.

## 4 Methodology

We estimate the forecasting model using a dynamic model averaging approach. We primarily use a direct (multi-step) forecast, and due to the dynamic model averaging framework, we use a recursive (expanding) window approach, i.e., every period, we add the actual realization of the exchange rate and other explanatory variables. This is due to the fact that the model averaging is done recursively, which means that the estimation procedure allows for different models to be selected in every period, and this probability is based on the posterior model probability criterion. At each step, an h-period-ahead forecast is produced, utilizing a distinct set of models corresponding to each forecasting horizon. In the out-of-sample forecasting exercise, the initial 72 periods, equivalent to six years, are employed as the training dataset. The forecast horizon ranges from one month to 36 months.^13^ We stress that in the out-of-sample forecasting exercise, the selection of explanatory variables and updating of the coefficients are also done in a (pseudo) real-time setting. More specifically, we estimate the following set of models (expressed in the state-space form) for every currency pair (see Equations (1) and (2)):

(1) $$\Delta_{t+h}e_{i,t}=A_{i,t}+B_{i,t}e_{i,t}+C_{i,t}f_{i,t}+\varepsilon_{i,t+h}$$

(2) $$\gamma_{i,t+1}=\gamma_{i,t}+\xi_{i,t}$$

where $e_{i,t}$ denotes chosen currency pair^14^ *i* (AUD/USD, CAD/USD, CHF/USD, EUR/USD, GBP/USD, JPY/USD, NOK/USD, NZD/USD, SEK/USD), $\Delta_{t+h}$ denotes the h step forward difference operator, and *h* is the number of leads, i.e., $\Delta_{t+h}e_{i,t}=e_{i,t+h}-e_{i,t}$.^15^ Furthermore, $e_{i,t}$ denote nominal exchange rate, $A_{i,t}$ is the constant term, and $B_{i,t},C_{i,t}$ are regression parameters, and $\varepsilon_{i,t+h}$ denotes stochastic term in observation equation following normal distribution and $var(\varepsilon_{i,t+h})=\sigma_{i,t+h,\varepsilon }^{2}$. We emphasize that $A_{i,t},B_{i,t},C_{i,t}$ are parameters that are time-varying. Furthermore, $f_{i,t}$ denotes the vector of other possible explanatory variables (fundamentals), i.e., $f_{i,t}$ is created from the following variables: $\left(m_{US},m_{domestic},p_{US},p_{domestic},un_{US},un_{domestic},ir_{US},ir_{domestic}\right)$, where index *US* denotes United States fundamentals and index domestic denotes the fundamentals of the domestic country related to the chosen currency pair *i*. In more detail, $m_{US}$ is the loans to non-financial corporations (money) in the US, and $m_{domestic}$ is the loans to (non-financial) corporations in the domestic country in the chosen currency pair. $p_{US}$ and $p_{domestic}$ denote the price level of countries, $un_{US}$ and $un_{domestic}$ are the unemployment rates regarding the chosen currency pair, $ir_{US}$ and $ir_{domestic}$ denote interest rates regarding the chosen currency pair. To simplify notation, we also introduced $\gamma_{i,t}=(A_{i,t},B_{i,t},C_{i,t})$, where $\gamma_{i,t}$ follows a random walk. Therefore, regarding state equation $\xi_{i,t}$ denotes the error term, and also set $var(\xi_{i,t})=\sigma_{i,t,\xi }^{2}$. The errors, $\varepsilon_{i,t+h}$ and $\xi_{i,t}$, are assumed to be mutually independent at all leads and lags.

Based on the economic theory described in the literature section, we can also a priori (and with a large amount of simplification) determine which signs we expect for the coefficients (or combination of coefficients) regarding fundamentals.^16^ The description below also focuses on coefficients regarding the medium and long-term forecasting horizon, where we believe there should be at least some convergence to a fundamental value. Furthermore, it should be noted that due to the importance of the US economy, variables regarding the US economy may act as proxies for general risk aversion. Based on purchasing power parity (PPP), we expect the coefficient regarding $p_{US}$ to be positive and the coefficient regarding $p_{domestic}$ to be negative. In other words, the currency *i* depreciates if the (domestic) country domestic has a higher price level than the US price level, i.e., $e_{i,t}=p_{US,t}-p_{domestic,t}$. Based on the monetary theory of exchange rates, we again expect the coefficient regarding $m_{US}$ to be positive and the coefficient regarding $m_{domestic}$ to be negative, as the model is an extension of the PPP theory. Based on the uncovered interest rate parity (UIRP) and standard formulation of monetary policy rules, we expect the coefficients regarding $un_{US}$ and $ir_{US}$ to be positive and the coefficients regarding $un_{domestic}$ and $ir_{domestic}$ to be negative. This is based on the following UIRP relation: $\Delta_{t+h}e_{i,t}=ir_{US,t}-ir_{domestic,t}$. However, an increase in the interest rate, whether current or expected in the future, should cause an immediate appreciation of the dollar, followed by forecasted (and actual) depreciation. For a comprehensive discussion on this topic, see, for example, [33], where authors argue for opposite signs regarding ($un_{US},un_{domestic}$) due to the (interest rate) smoothing. Also, previous studies, for example by [61], have also shown that carry trades are generally profitable, which suggests the invalidity of uncovered interest rate parity in the medium and short run.^17^ Furthermore, since the fundamental variables enter the model separately, we do not restrict their coefficients, and given the effect of, for example, risk premium, we do not expect cointegration vectors of exactly (1,−1) for any of the above relation.

Note that specification (1) represents the most parsimonious form, where we do not impose a cointegration relation a priori; rather, it is implicitly accounted for in the estimation process. To illustrate this, the expression can be rewritten as follows, where $c_{i,t},d_{i,t}$ are the new constant terms, and $\gamma_{i,t},\delta_{i,t}$ are the new regression parameters: $\Delta_{t+h}e_{i,t}=c_{i,t}+\delta_{i,t}(e_{i,t}-\gamma_{i,t}f_{i,t}+d_{i,t})+\varepsilon_{i,t+h}\;$ This specification (error correction model) is often used to calculate forecasts based on the monetary and Taylor rule models.^18^ As [17] points out, this specification is a projection of the h-step change in the nominal exchange rate on the current deviation from the fundamental value. In summary, each time series regarding the explanatory variables enters the model separately as we do not impose cointegration vectors a priori.

As mentioned above, the approach used for the estimation is dynamic model averaging (DMA), which is a relatively novel approach in exchange rate forecasting literature. This approach uses a set of approximations to evaluate a large number of models in every period, which significantly reduces the computational burden. In short, the DMA approach combines the merits of time-varying parameter models with the Bayesian model averaging framework. This is especially suitable for this forecasting exercise given a large uncertainty about which set of variables best characterizes the development of exchange rates. Moreover, we also suspect that the relevance of individual variables or combinations may vary over time, for example, due to different economic conditions over the business and financial cycle. Similar to the argument by [13], we consider this a natural way to reduce instability problems. More specifically, dynamic model averaging (DMA) uses sets of approximations regarding Kalman filter equations and Markov-switching models to reduce computational complexity. DMA framework primarily builds upon the Kalman filter equations for the estimation of individual models, but the prediction formula for the variance in the Kalman filter is simplified.^19^ In more detail, the traditional prediction formula for the variance of the prediction error can be written as follows (see Equation (3)):

(3) $$P_{i,t|t-1}=P_{i,t-1|t-1}+\sigma_{i,t,\xi }^{2}$$

The formula can be simplified (replaced) in the following way using forgetting factor *λ* (see Equation (4)):

(4) $$P_{i,t|t-1}=\frac{1}{\lambda_{i,t}}P_{i,t-1|t-1}$$

where the $\lambda_{i,t}$ regulates the uncertainty regarding the state (i.e. time-varying coefficient) evolution, and it is typically set slightly below 1. (Values close to 1 lead to a more stable model.) Therefore, we can write the following (see Equation (5)):

(5) $$\sigma_{i,t,\xi }^{2}=(1-\frac{1}{\lambda_{i,t}})P_{i,t-1|t-1}$$

We also follow [62], [63] and estimate the forgetting factor $\lambda_{i,t}$ as a time-varying parameter. This forgetting factor used in approximation regarding the prediction error variance $\lambda_{i,t}$ is set to be a time-varying parameter between 0.9 and 1. More specifically, we estimate $\lambda_{i,t}$ in following way (see Equation (6)):

(6) $$\lambda_{i,t}=\lambda_{min}+(1-\lambda_{min})L^{f_{i,t}}$$

$\lambda_{min}$ denotes the minimum value of the forgetting factor, and L defines the sensitivity of the coefficients' variation to prediction errors. We set $\lambda_{min}=0,9$ and Ł=1,1 and we further define $f_{t}$ as one-step-ahead prediction error squared. $\sigma_{i,t+h,\varepsilon }^{2}$ is then obtained using an exponentially weighted moving average estimate, which can be written as follows (see Equation (7)):

(7) $$\sigma_{i,t+h,\varepsilon }^{2}=\kappa \sigma_{i,t+h-1,\varepsilon }^{2}+(1-\kappa )\varepsilon_{i,t+h}^{2}$$

where *κ* denotes another decay factor used in the DMA framework, which is set to a value of 0.96. However, the results were generally robust to different specifications between 0.9-0.98. We also use diffuse priors to initialize the Kalman filter recursion. Another set of approximations is to avoid specifying the transition matrix between different model specifications. The simplification is based on replacing the traditional transition matrix by introducing another forgetting factor *λ*, which controls the ‘speed of model-switching’. The following approximation replaces the model prediction equation (see Equation (8)):

(8) $$\pi_{i,t|t-1,k}=\frac{\pi_{i,t-1|t-1,k}^{\alpha }}{\sum_{l=1}^{l=M}\pi_{i,t-1|t-1,l}^{\alpha }}$$

where $\pi_{i,t|t-1,k}$ denotes the probability of model *k* being the correct one at time *t*. We also set the forgetting factor *λ* in the approximation to the value 0.95,^20^ and *M* is the number of competing models. In other words, we estimate a set of *M* models, which are characterized by having different subsets of predictors. There are $2^{K}-1$ possible models to construct, where *K* denotes all possible explanatory variables (including constant term). Note, however, that in some of the models considered in the DMA framework, the economic theories of exchange rate determination may occur in some combinations, but other models will not correspond to such concepts. We could easily restrict the models considered and allow a priori only a subset of these models based on economic theories. We, however, prefer to rely on a more agnostic approach regarding the predictor variables and include all possible combinations. Alternatively, we can consider these (non-stationary) variables a proxy for drift/trend.^21^

In our forecasting exercise, we thus evaluate all possible combinations of explanatory variables. Updated model probabilities can then be written as follows (see Equation (9)):

(9) $$\pi_{i,t|k}^{t-1}=\frac{p_{k}(\Delta_{t+h}e_{i,t}|\Delta_{t+h}e_{i}^{t-1})\pi_{i,t|t-1,k}^{\alpha }}{\sum_{l=1}^{l=M}p_{l}(\Delta_{t+h}e_{i,t}|\Delta_{t+h}e_{i}^{t-1})\pi_{i,t|t-1,l}^{\alpha }}$$

where $p_{l}(\Delta_{t+h}e_{i,t}|\Delta_{t+h}e_{i}^{t-1})$ denote the predictive density for model *l* regarding currency *i* obtained by the Kalman filter. We set $\pi_{i,0|0,k}$ to 1/M, which can be considered un-informative prior. A significant advantage of this approach is that, due to the use of a set of approximations, it is quite easy to calculate the posterior inclusion probabilities on individual models, i.e., all possible combinations of explanatory variables. The out-of-sample forecasting is done by employing the prediction equation from the Kalman filter and the posterior model probabilities, which are lagged by one period to avoid ‘data snooping’. For more technical details regarding dynamic model averaging, see [62], [64], or [63].

The predictive accuracy was assessed through several methods. Firstly, we compared the root-mean-square forecast error (RMSFE) of the DMA model to that of a random walk benchmark, where the random walk without drift generates forecasts according to: ${\overset{ˆ}{e}}_{t+h}=e_{t}$. The model is considered superior to the random walk benchmark if the RMSFE ratio is less than one. Additionally, we utilized the mean absolute forecast error (MAFE) as a comparison metric. MAFE is included because it assigns less weight to large forecast errors compared to RMSFE. The interpretation of the MAFE ratio is analogous to that of the RMSFE ratio. In the robustness check section, we replace the random walk benchmark with an alternative benchmark based on OLS regression.

We also utilized the test statistics (DMW) proposed by [65] for a more formal evaluation. This test examines the null hypothesis that the DMA model's root-mean-square forecast error (RMSFE) is equal to that of the random walk (or alternative OLS) benchmark. The alternative hypothesis posits that the DMA model outperforms the benchmark model. Additionally, we employed the test statistics (CW) developed by [66].^22^ [66] demonstrate that the asymptotic distributions of the sample difference between two RMSFE ratios are not identical and are biased downward from zero. This adjustment accounts for the fact that when comparing two models, where one model is nested within the other, the nested model has fewer parameters to estimate, leading to reduced estimation error. However, it is important to note that our recursive approach does not fully satisfy all the assumptions required by these tests.

## 5 Empirical results

The results of our forecasting exercise are presented in Table 1, which is organized by currency pairs.^23^ The first row reports the ratio of the root mean square forecast error (RMSFE) of the DMA forecast relative to the random walk benchmark across various time horizons. The second row provides the same ratio, but for the mean absolute forecast error (MAFE). The third and fourth rows display p-values for the Diebold-Mariano (DMW) and Clark-West (CW) test statistics, respectively. P-values for these test statistics are based on a one-sided test with the alternative hypothesis that the specified model outperforms the random walk. In the final row, the number of forecast periods is indicated. The individual columns (Mx) represent the forecast results for x months ahead.

**Table 1:** Out of sample forecast evaluation - random walk benchmark.

AUD/USD	M1	M3	M6	M9	M12	M24	M36
RMSFE_M/RMSFE_RW	1.01	0.98	0.89	0.82	0.79	0.62	0.54
MAFE_M/MAFE_RW	1.00	0.98	0.92	0.90	0.86	0.79	0.72
DMW	0.70	0.37	0.01	0.00	0.01	0.00	0.00
CW	0.09	0.01	0.00	0.00	0.00	0.00	0.00
N	239	237	234	231	228	216	204

**CAD/USD**	M1	M3	M6	M9	M12	M24	M36

RMSFE_M/RMSFE_RW	1.01	0.99	0.95	0.90	0.88	0.70	0.63
MAFE_M/MAFE_RW	1.00	1.00	0.97	0.95	0.92	0.84	0.80
DMW	0.89	0.39	0.12	0.01	0.03	0.04	0.02
CW	0.60	0.03	0.00	0.00	0.00	0.00	0.00
N	239	237	234	231	228	216	204

**CHF/USD**	M1	M3	M6	M9	M12	M24	M36

RMSFE_M/RMSFE_RW	1.03	1.10	1.06	0.92	0.78	0.57	0.51
MAFE_M/MAFE_RW	1.01	1.04	1.00	0.96	0.90	0.77	0.70
DMW	0.97	0.99	0.76	0.20	0.06	0.08	0.13
CW	0.91	0.94	0.19	0.01	0.01	0.01	0.03
N	179	177	174	171	168	156	144

**EUR/USD**	M1	M3	M6	M9	M12	M24	M36

RMSFE_M/RMSFE_RW	1.04	1.04	0.87	0.81	0.69	0.60	0.70
MAFE_M/MAFE_RW	1.01	1.01	0.92	0.88	0.83	0.76	0.81
DMW	0.98	0.80	0.04	0.04	0.01	0.03	0.12
CW	0.95	0.21	0.00	0.00	0.00	0.01	0.05
N	167	165	162	159	156	144	132

**GBP/USD**	M1	M3	M6	M9	M12	M24	M36

RMSFE_M/RMSFE_RW	1.02	1.01	0.90	0.79	0.71	0.58	0.56
MAFE_M/MAFE_RW	1.01	1.00	0.94	0.90	0.86	0.78	0.76
DMW	0.96	0.57	0.08	0.05	0.03	0.04	0.01
CW	0.81	0.06	0.00	0.00	0.00	0.01	0.01
N	231	229	226	223	220	208	196

**JPY/USD**	M1	M3	M6	M9	M12	M24	M36

RMSFE_M/RMSFE_RW	1.04	1.03	0.90	0.78	0.71	0.50	0.37
MAFE_M/MAFE_RW	1.02	1.03	0.96	0.88	0.83	0.69	0.61
DMW	0.96	0.73	0.09	0.03	0.01	0.02	0.02
CW	0.67	0.14	0.00	0.00	0.00	0.00	0.00
N	194	192	189	186	183	171	159

**NOK/USD**	M1	M3	M6	M9	M12	M24	M36

RMSFE_M/RMSFE_RW	1.02	1.03	0.96	0.88	0.84	0.71	0.66
MAFE_M/MAFE_RW	1.01	1.00	0.96	0.91	0.91	0.86	0.81
DMW	0.97	0.78	0.27	0.04	0.07	0.03	0.09
CW	0.74	0.13	0.00	0.00	0.00	0.00	0.00
N	239	237	234	231	228	216	204

**NZD/USD**	M1	M3	M6	M9	M12	M24	M36

RMSFE_M/RMSFE_RW	1.02	1.01	0.97	0.92	0.83	0.75	0.76
MAFE_M/MAFE_RW	1.01	1.00	1.00	0.95	0.91	0.82	0.87
DMW	0.86	0.58	0.21	0.02	0.04	0.04	0.03
CW	0.51	0.03	0.00	0.00	0.00	0.00	0.00
N	222	220	217	214	211	199	187

**SEK/USD**	M1	M3	M6	M9	M12	M24	M36

RMSFE_M/RMSFE_RW	1.04	1.04	0.91	0.86	0.77	0.61	0.62
MAFE_M/MAFE_RW	1.01	1.01	0.95	0.91	0.88	0.75	0.78
DMW	0.92	0.78	0.01	0.00	0.01	0.03	0.05
CW	0.71	0.25	0.00	0.00	0.00	0.00	0.01
N	180	178	175	172	169	150	140

***Note:*** The dependent variable is the h-step forward difference of the given currency pair. The individual columns (Mx) represent the results for the x month ahead forecast. RMSFE_M/RMSFE_RW is the ratio of root mean squared forecast error of the DMA framework and random walk benchmark. MAFE_M/MAFE_RW represents the ratio of the mean absolute forecast error between the two. DMW and CW indicate the p-values for the Diebold-Mariano and Clark-West test statistics, respectively. N refers to the number of estimated forecasts. DMA framework was used for the estimation.

Our main findings are as follows: The DMA approach clearly defeats the random walk benchmark based on the RMSFE and MAFE ratio in the medium and long-term horizon. This result is generally robust as it applies to all exchange rate pairs. Regarding the period of the first six months, the random walk benchmark and DMA forecasts are approximately equal in terms of forecasting. There are, of course, some differences in forecasting power regarding different currencies. It can be summarized that the success of the various estimated models varies depending on the chosen forecast horizon and exchange rate. Overall, the results provide substantial support for our assertion of significant nominal exchange rate predictability in the medium to long term.

In more detail, we find that no model outperforms the random walk at the one-month horizon. The same applies for the three-month horizon with the exception of the AUD/USD and NZD/USD currency pairs. These short-term forecasting results align with the existing literature, reinforcing the notion that it is exceedingly difficult to identify models that consistently outperform the random walk benchmark when evaluated using standard RMSFE and MAFE criteria. However, as previously noted, the forecasting performance according to the RMSFE and MAFE ratios does not significantly deviate from the random walk benchmark. Beyond the six-month horizon, the DMA approach surpasses the random walk benchmark across all currencies, as indicated by the RMSFE, MAFE ratios, and CW statistics. Also, according to DMW statistics, the DMA approach is statically distinguishable from the random walk benchmark in most cases. For example, in 12 month period, except for the CHF/USD currency pair, DMA provides better predictions according to DMW statistics at the five percent significance level.

Furthermore, we observe strong predictability at longer forecast horizons, consistent with findings by [4], particularly at the two- and three-year horizons. This is evidenced by the declining RMSFE and MAFE ratios, indicating increasing predictability as the forecast horizon extends. In this context, the DMA predictions outperform the random walk benchmark significantly, as reflected in the RMSFE and MAFE ratios. The results are further corroborated by the CW test statistics, where, in most cases, the CW test distinguishes between the DMA forecast and the random walk benchmark at a five percent significance level. The results are, however, a bit more mixed according to DMW statistics, but in a majority of cases, still within a five percent significance level. For comparison, the long-term forecast results are on par with, and in some cases superior to, those found in existing studies on nominal exchange rate forecasting (e.g., [36], [67]) at the three-year forecast horizon. This suggests that the DMA delivers outstanding forecasting performance in the very long run.

**Figure B.1:**
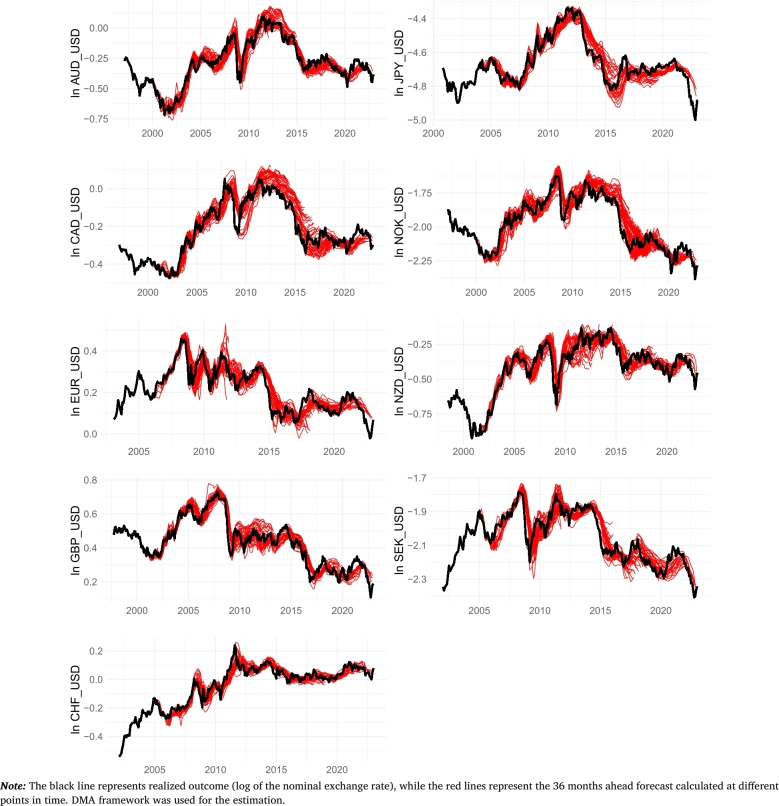
Out of sample sequential forecast.

Fig. B.1 in the Appendix provides further intuition on the results obtained.^24^ While it is generally challenging to evaluate the forecast quality solely through the chart, we view Fig. B.1 as a supplementary tool to Table 1. The chart allows for an assessment of forecast quality across different time periods. Nevertheless, it is evident that the forecast consistently outperforms the random walk in the long term. More importantly, we can observe that the prediction accuracy is approximately constant over the period considered, which suggests that the model can consistently handle forecasting under different economic conditions, and the results are not driven by good forecasting performance in only a subset of periods.

How should we interpret the results obtained, given that the nominal exchange rate seems predictable contrary to much of the forecasting literature? We offer several possible explanations. One possible explanation is that by using a large number of variables, we implicitly identify the cointegration vector, and the nominal exchange rate converges to the equilibrium value defined by this vector. However, this result contrasts with the theoretical finding by [42] because the explanatory variables are highly persistent, and the discount factor is close to one. An alternative explanation can be based on the ‘scapegoat theory of exchange rates’ or on the claim that the forecasts obtained are, in reality, rooted in the stationarity and predictability of risk premia, which is indirectly estimated by the fundamental factors (explanatory variables). Another explanation is that the (nominal) exchange rates are mean-reverting. This reasoning aligns with [36], where authors present a similar argument for real exchange rates. It seems reasonable that even the nominal exchange rates can be considered stationary in the period examined, which is characterized by mostly low inflation rates and central banks targeting an explicitly set inflation target.^25^ It should also be noted that these possible explanations are not mutually exclusive. The nominal exchange rate may be stationary, but other variables may help determine its (fundamental) value.

Furthermore, we believe the second crucial modelling decision contributing to the observed nominal exchange rate predictability in the medium and long term is the adoption of direct forecasting. This approach accounts for the fact that exchange rate dynamics can be obscured by high-frequency ‘non-fundamental’ noise, which is inherently difficult to predict. By effectively filtering out this noise, a model may demonstrate strong predictive capability over the medium to long term, precisely because it avoids targeting unpredictable high-frequency fluctuations. Consequently, we prefer to utilize direct forecasting, where prediction errors do not accumulate. In contrast, with recursive forecasting, a minor perturbation in one period's forecast can lead to substantial deviations in predictions over subsequent periods.

Which of the proposed explanations for exchange rate predictability can be considered the most likely? The dynamic model averaging (DMA) approach provides us with posterior model probability, allowing us to access the importance of individual variables, which can help us identify the reason for exchange rate predictability. That being said, it can be reasonably expected that the variables selected the most (with the highest posterior inclusion probability^26^) are the reason behind the exchange rate predictability. Table 2 displays the posterior probability of inclusion of the given explanatory variable. Given that probability is time-varying, we show the median value over the whole horizon (not including the training period). The results also indicate fast model switching (not shown). Table 2 indicates that the variable with the highest posterior probability of being included is the nominal exchange rate value. The probability of including any other explanatory variable is significantly lower. The probability of including a nominal exchange rate in estimation is highest in the medium and long run, i.e., in periods where the model performs the best.^27^ The results support the claim that nominal exchange rates are mean reverting in the period under consideration. On the other hand, this in itself does not mean that the lagged exchange rate is the only and best predictor for predicting changes in exchange rates. It follows from the definition of the co-integration vector, i.e., if we were able to identify the deviation from fundamental value, that even in this case, the exchange rate should be included. Therefore, most economically reasonable specifications include a lagged exchange rate as an explanatory variable.

**Table 2:** Posterior inclusion probability.

AUD/USD	M1	M3	M6	M9	M12	M24	M36
AUD/USD	0.53	0.73	0.97	1.00	1.00	1.00	1.00
US money	0.44	0.52	0.76	0.85	0.87	0.89	0.55
AUD money	0.48	0.57	0.68	0.79	0.74	0.73	0.78
US unemployment	0.48	0.47	0.49	0.55	0.49	0.48	0.35
AUD unemployment	0.47	0.50	0.47	0.43	0.41	0.48	0.46
US price level	0.44	0.48	0.54	0.51	0.49	0.35	0.44
AUD price level	0.43	0.50	0.53	0.46	0.46	0.41	0.35
US interest rate	0.46	0.44	0.45	0.53	0.46	0.22	0.48
CA interest rate	0.45	0.45	0.52	0.56	0.40	0.50	0.71
constant	0.45	0.48	0.54	0.52	0.48	0.44	0.42

**CAD/USD**	M1	M3	M6	M9	M12	M24	M36

CAD/USD	0.53	0.72	0.86	0.92	0.94	0.97	0.98
US money	0.46	0.49	0.52	0.51	0.48	0.41	0.31
CA money	0.48	0.56	0.52	0.54	0.47	0.30	0.31
US unemployment	0.44	0.50	0.49	0.50	0.50	0.43	0.38
CA unemployment	0.44	0.47	0.47	0.47	0.37	0.31	0.38
US price level	0.48	0.52	0.54	0.54	0.46	0.47	0.42
CA price level	0.47	0.48	0.49	0.46	0.44	0.46	0.42
US interest rate	0.46	0.49	0.40	0.33	0.28	0.13	0.15
CA interest rate	0.47	0.46	0.48	0.45	0.51	0.45	0.46
constant	0.42	0.51	0.53	0.54	0.54	0.57	0.66

**CHF/USD**	M1	M3	M6	M9	M12	M24	M36

CHF/USD	0.58	0.86	0.97	1.00	1.00	1.00	0.99
US money	0.44	0.43	0.53	0.54	0.56	0.70	0.40
CH money	0.44	0.48	0.50	0.46	0.35	0.59	0.46
US unemployment	0.33	0.44	0.50	0.50	0.60	0.64	0.40
CH unemployment	0.45	0.49	0.40	0.41	0.40	0.44	0.38
US price level	0.45	0.52	0.71	0.82	0.91	0.53	0.37
CH price level	0.45	0.44	0.50	0.37	0.39	0.49	0.61
US interest rate	0.42	0.37	0.22	0.22	0.21	0.66	0.28
CH interest rate	0.42	0.43	0.44	0.43	0.39	0.57	0.33
constant	0.46	0.55	0.65	0.76	0.88	0.60	0.84

**EUR/USD**	M1	M3	M6	M9	M12	M24	M36

EUR/USD	0.55	0.84	0.96	1.00	1.00	1.00	0.69
US money	0.37	0.40	0.44	0.47	0.36	0.61	0.49
EU money	0.36	0.45	0.68	0.67	0.66	0.70	0.40
US unemployment	0.27	0.45	0.52	0.51	0.50	0.44	0.70
EU unemployment	0.39	0.38	0.39	0.32	0.44	0.27	0.38
US price level	0.40	0.46	0.52	0.59	0.53	0.43	0.41
EU price level	0.40	0.45	0.49	0.50	0.50	0.44	0.40
US interest rate	0.37	0.38	0.48	0.55	0.65	0.91	0.22
EU interest rate	0.36	0.38	0.43	0.30	0.39	0.20	0.28
constant	0.43	0.45	0.42	0.40	0.38	0.38	0.49

**GBP/USD**	M1	M3	M6	M9	M12	M24	M36

GBP/USD	0.50	0.70	0.89	0.98	0.99	1.00	1.00
US money	0.44	0.60	0.85	0.93	0.94	0.91	0.50
GB money	0.39	0.49	0.38	0.36	0.39	0.39	0.41
US unemployment	0.44	0.43	0.32	0.38	0.38	0.44	0.53
GB unemployment	0.40	0.37	0.26	0.21	0.32	0.43	0.57
US price level	0.42	0.47	0.44	0.38	0.67	0.60	0.59
GB price level	0.42	0.51	0.44	0.41	0.46	0.51	0.59
US interest rate	0.43	0.43	0.41	0.40	0.37	0.41	0.44
GP interest rate	0.43	0.43	0.40	0.37	0.39	0.40	0.38
constant	0.42	0.47	0.44	0.44	0.46	0.44	0.50

**JPY/USD**	M1	M3	M6	M9	M12	M24	M36

JPY/USD	0.48	0.83	0.97	0.95	0.98	1.00	1.00
US money	0.41	0.38	0.40	0.45	0.44	0.30	0.25
JPN money	0.41	0.46	0.48	0.52	0.61	0.54	0.72
US unemployment	0.39	0.38	0.35	0.34	0.32	0.40	0.37
JPN unemployment	0.39	0.35	0.25	0.28	0.29	0.45	0.45
US price level	0.42	0.41	0.46	0.41	0.43	0.38	0.38
JPN price level	0.43	0.44	0.46	0.44	0.41	0.47	0.52
US interest rate	0.42	0.35	0.25	0.31	0.30	0.37	0.46
JPN interest rate	0.42	0.41	0.49	0.74	0.45	0.75	0.52
constant	0.43	0.48	0.50	0.46	0.49	0.55	0.55
**NOK/USD**	M1	M3	M6	M9	M12	M24	M36

NOK/USD	0.51	0.72	0.90	0.97	0.98	0.98	0.82
US money	0.40	0.51	0.70	0.79	0.89	0.41	0.35
NO money	0.44	0.49	0.59	0.56	0.60	0.55	0.34
US unemployment	0.39	0.35	0.33	0.34	0.40	0.42	0.64
NO unemployment	0.45	0.41	0.33	0.41	0.38	0.42	0.45
US price level	0.44	0.46	0.46	0.39	0.46	0.53	0.36
NO price level	0.43	0.46	0.44	0.44	0.47	0.38	0.40
US interest rate	0.43	0.43	0.31	0.39	0.39	0.43	0.21
NO interest rate	0.43	0.43	0.31	0.39	0.39	0.43	0.21
constant	0.44	0.46	0.51	0.45	0.40	0.48	0.67

**NZD/USD**	M1	M3	M6	M9	M12	M24	M36

NZD/USD	0.53	0.70	0.85	0.96	0.99	1.00	1.00
US money	0.43	0.53	0.44	0.61	0.65	0.81	0.63
NZD money	0.45	0.45	0.45	0.42	0.44	0.46	0.54
US unemployment	0.42	0.46	0.42	0.42	0.40	0.55	0.58
NZD unemployment	0.44	0.44	0.40	0.39	0.35	0.62	0.48
US price level	0.45	0.51	0.54	0.50	0.61	0.53	0.46
NZD price level	0.45	0.51	0.45	0.45	0.57	0.43	0.47
US interest rate	0.42	0.46	0.44	0.52	0.81	0.73	0.53
NZD interest rate	0.46	0.46	0.46	0.38	0.42	0.61	0.52
constant	0.45	0.51	0.46	0.43	0.49	0.85	0.84

**SEK/USD**	M1	M3	M6	M9	M12	M24	M36

SEK/USD	0.48	0.65	0.83	0.98	0.99	1.00	0.66
US money	0.39	0.42	0.65	0.60	0.51	0.49	0.41
SWE money	0.42	0.48	0.48	0.43	0.52	0.45	0.45
US unemployment	0.39	0.41	0.46	0.57	0.52	0.42	0.53
SWE unemployment	0.44	0.43	0.43	0.47	0.47	0.43	0.35
US price level	0.43	0.50	0.53	0.48	0.58	0.45	0.50
SWE price level	0.41	0.48	0.47	0.48	0.52	0.53	0.51
US interest rate	0.38	0.38	0.46	0.48	0.58	0.50	0.32
SWE interest rate	0.37	0.42	0.44	0.41	0.37	0.38	0.42
constant	0.41	0.41	0.46	0.50	0.77	0.58	0.53

***Note:*** The table displays variable inclusion probability for different explanatory variables. The individual columns (Mx) denote the results for the x month ahead forecast. DMA framework was used for the estimation.

Interestingly, explanatory variables that act as a proxy for money also have a fairly high probability of being included in the estimates, especially in the medium and long-term horizon. This suggests that money, specifically loans to corporations, which act as a proxy for corporate deposits, may also play a role in determining the exchange rate. This indicates some validity of the monetary model, i.e., either through the determination of the fundamental value or through the indirect estimation of the risk premia or as a ‘scapegoat’ variable.^28^ Price levels, interest rates, and unemployment rates have somewhat lower inclusion probability than explanatory variables that act as a proxy for money. Regarding price levels, a possible explanation is the stable rate of inflation in the period and countries examined. Regarding unemployment rates and interest rates, these results suggest the invalidity of forecasting exchange rates based strictly on monetary policy rules or uncovered interest rate parity. On the other hand, we remind the readers that we work only with the data in (log) levels; a possible extension is to include growth rates or gap estimates.

We also expect that the coefficients regarding explanatory variables that are most stable over time are most likely to show a true relation. Gradual change within an (economic or financial) cycle is understandable, but coefficients characterized by high variability probably capture mostly noise than anything meaningful. Alternatively, the possible explanation of parameter instability is offered by the ‘scapegoat theory of exchange rates’, i.e., the existence of significant variation in the parameters is due to the fact that these variables act as ‘scapegoats’ and the true relationship is non-existent. For this reason, we examine the stability of the regression coefficients. Given the fact that we use a large number of explanatory variables, the time-varying nature of these parameters, and the general number of models estimated, we present the results as charts that display the median value and 25%–75% quantiles of the estimated regression coefficients. In more detail, the solid line represents the median value, and the dashed lines represent the 25%–75% quantiles.

**Figure 1:**
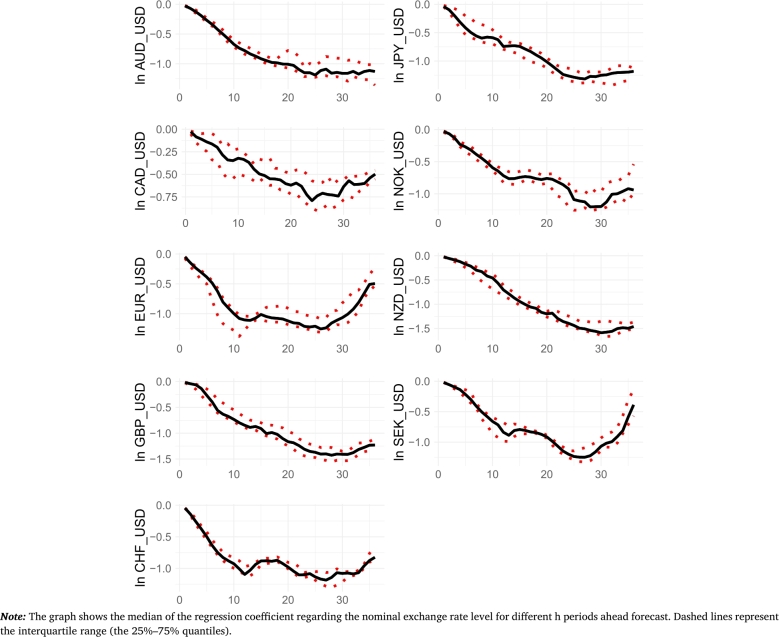
Regression coefficient - nominal exchange rate.

Firstly, we focus on the parameter related to the (lagged) value of the nominal exchange rate. This is because, as mentioned before, it is the most likely for this explanatory variable to be included in our forecasts. Fig. 1 displays the regression coefficients regarding the spot nominal exchange rate level for different forecasting horizons. The coefficients are clearly decreasing with the increasing time horizon of the forecast. These results suggest that there exists a positive auto-correlation in nominal exchange rates, which, however, can turn slightly negative for certain currencies (and only in the long run). It can be observed that the values of this coefficient in most cases do not differ much from the value implied by nominal exchange rates behaving according to an AR(1) process. To see this fact, equation (8) can be rewritten as follows: $e_{i,t+h}=A_{i}+(1+B_{i})e_{i,t}+C_{i}f_{i,t}+\varepsilon_{i,t+h}$. Furthermore, given that the value of these coefficients for the forecast for one period ahead is close to zero in all cases, a recursive forecast implies very different long-run forecast values given small perturbations in the parameter estimate. Moreover, while there is some variability and instability in the parameter estimates, we find the results encouraging, as the parameters do not exhibit dramatic fluctuations. Upon examining the time-varying nature of these parameters (not shown), the movements generally appear to be gradual. This may reflect shifts in some underlying economic processes.

**Figure B.2:**
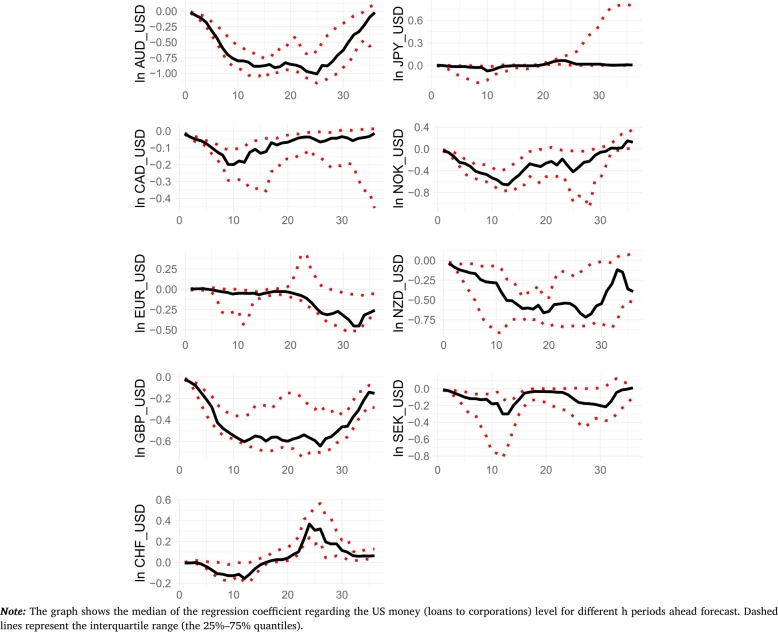
Regression coefficient - US money.

**Figure B.3:**
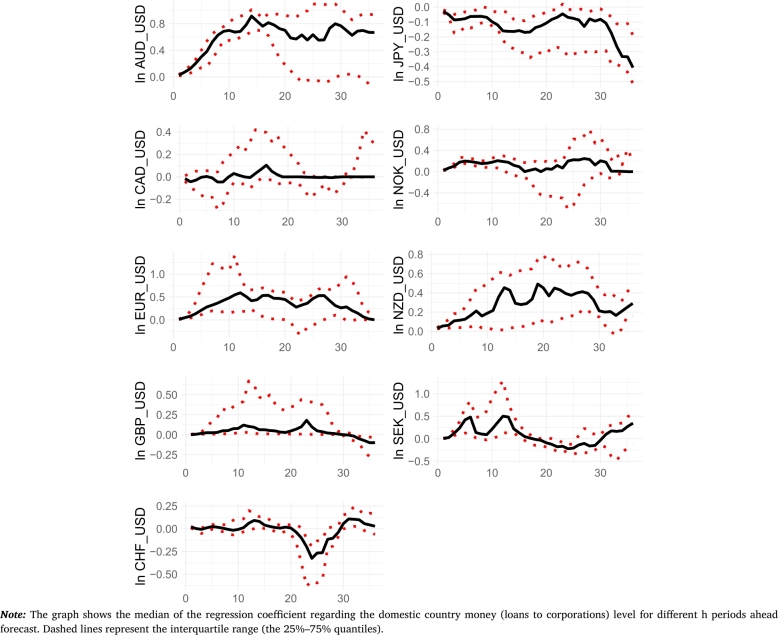
Regression coefficient - domestic country money.

**Figure B.4:**
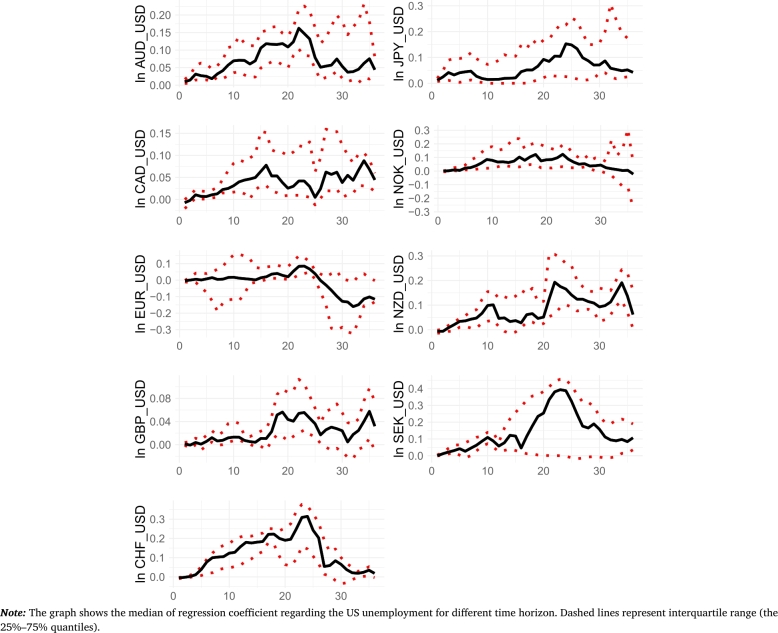
Regression coefficient - US unemployment.

**Figure B.5:**
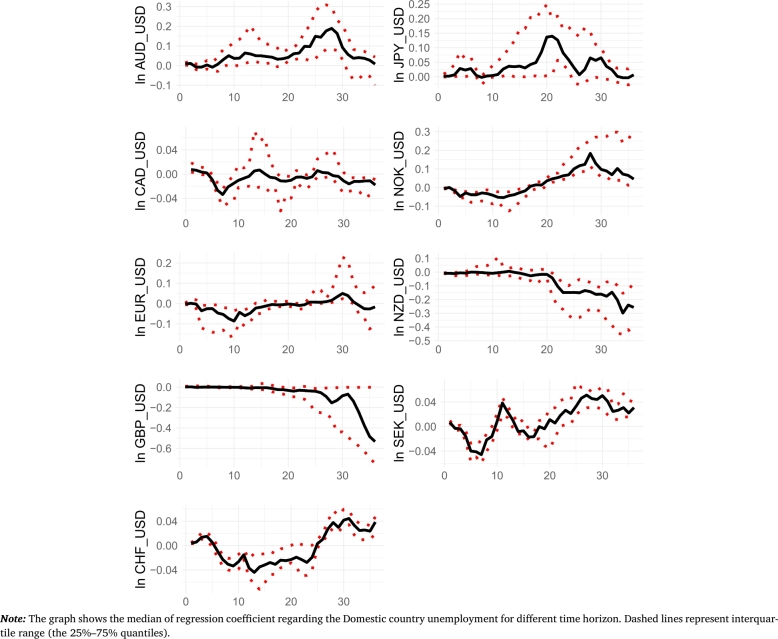
Regression coefficient - domestic country unemployment.

**Figure B.6:**
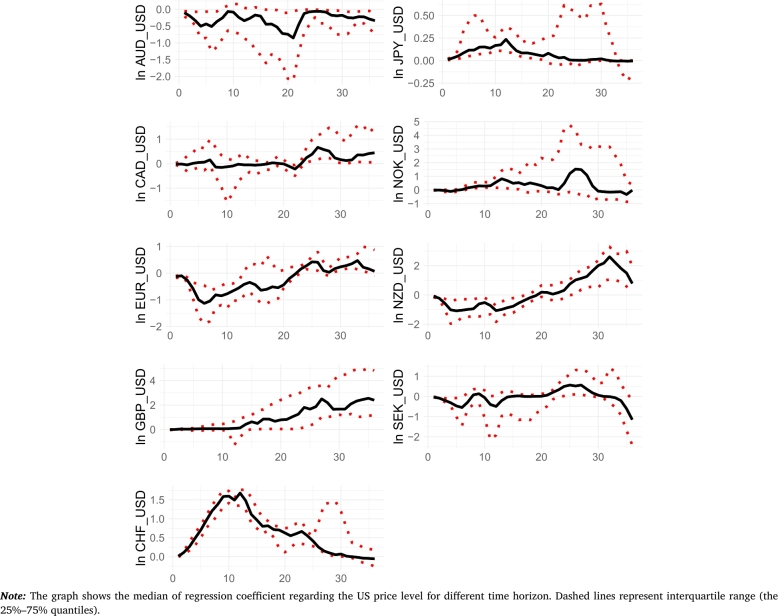
Regression coefficient - US price level.

**Figure B.7:**
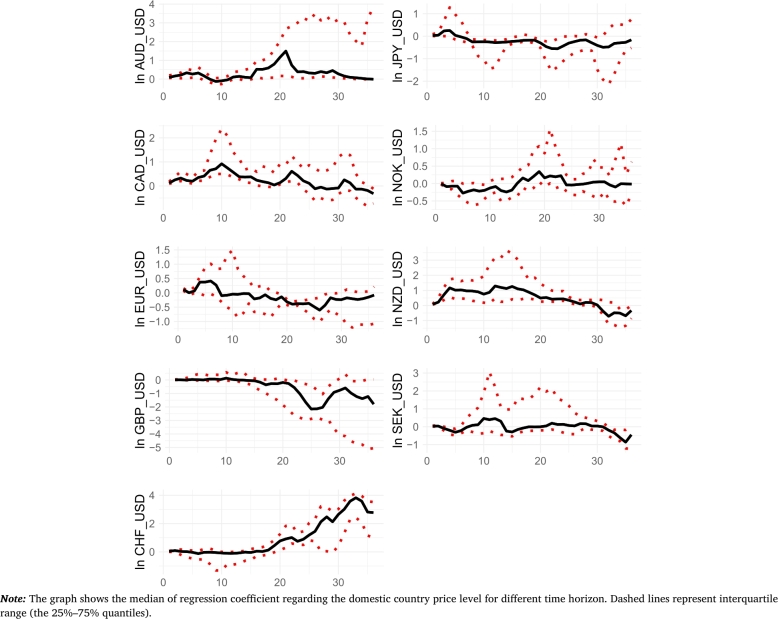
Regression coefficient - domestic country price level.

**Figure B.8:**
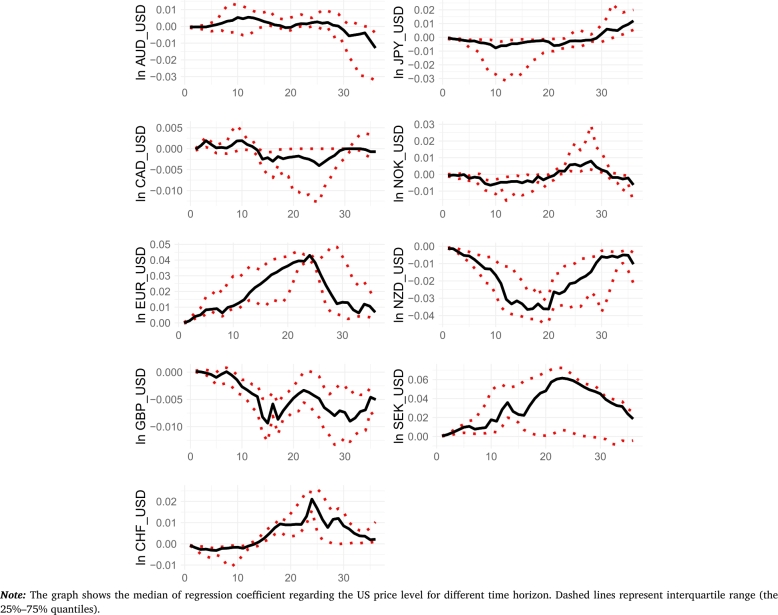
Regression coefficient - US interest rate.

**Figure B.9:**
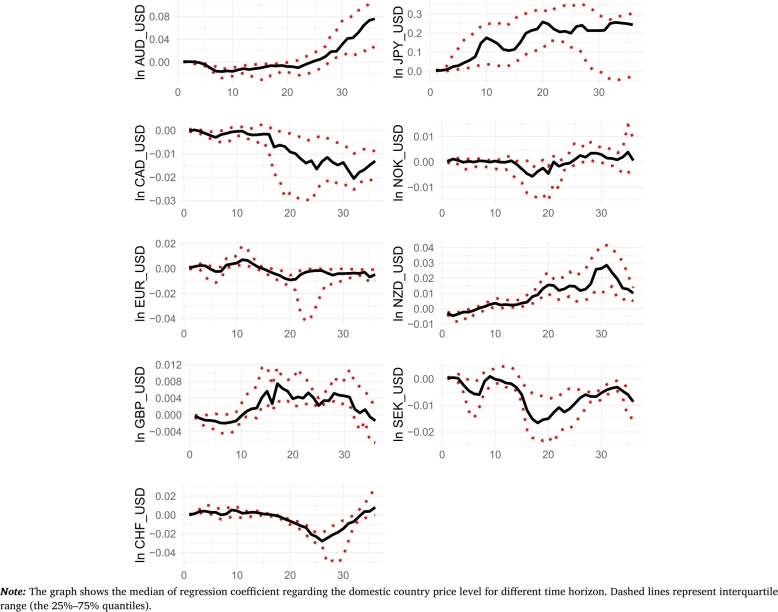
Regression coefficient - domestic country interest rate.

**Figure B.10:**
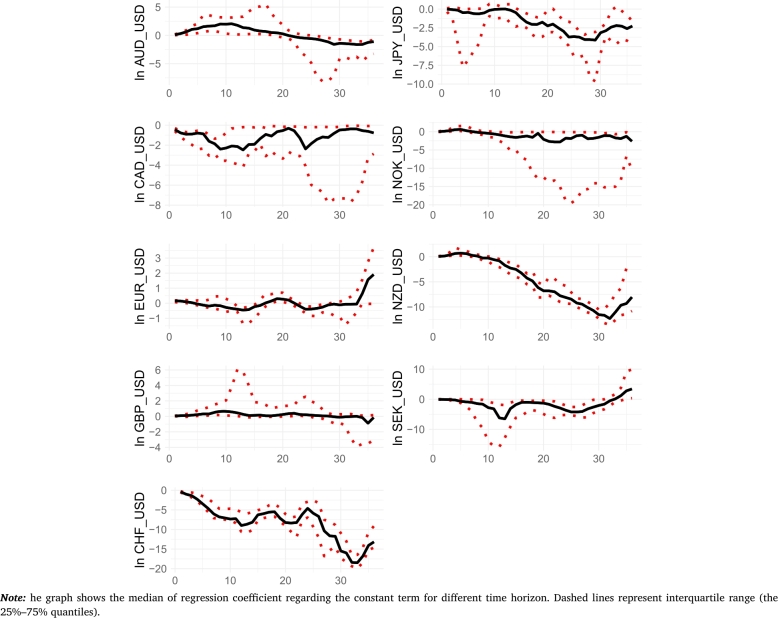
Regression coefficient - constant term.

For other variables, see Figure B.2, Figure B.3, Figure B.4, Figure B.5, Figure B.6, Figure B.7, Figure B.8, Figure B.9, Figure B.10 in the Appendix which display the regression coefficients regarding these variables. In general, these coefficients exhibit a much higher degree of instability compared to the coefficient for the (lagged) value of the nominal exchange rate. In many cases, the range of 25%–75% quantiles of these coefficients includes the zero value. This is especially true for short-term forecasts, highlighting the instability of these coefficients. Even when the median values of these coefficients for fundamentals are different from zero, the quantiles indicate high instability in the estimates. Furthermore, the coefficients for the same variable often differ across countries, i.e., the strength of the link between exchange rates and fundamentals varies across currencies. In other words, the relationship between fundamentals and exchange rates is not stable over time and often varies across currencies. As the forecast horizon lengthens, the coefficients (both median and 25%–75% quantiles) are more often different from zero, suggesting that these variables can have additional explanatory power in certain forecasting horizons.

In more detail, the coefficients regarding the price levels seem to be very close to zero in the short and medium run, and we again observe the issue that the coefficient signs of these variables indicate different relationships for different currencies. In the long run, they are distinguishable from zero and mostly have the correct sign. This suggests a very slow pass-through of price levels to the nominal exchange rate and, therefore, some validity of the PPP theory. In the case of the coefficients regarding the credit to corporations (a proxy for money), we can observe that these variables have mostly non-zero values, especially in the medium and long run. The issue is that the coefficient signs of these variables indicate different relationships for different currencies, and in many cases, the sign of these coefficients is opposite to what the theory implies. As explained in the methodology section, we expect the coefficients $m_{US}$ and $p_{US}$ to be positive and the coefficients $m_{domestic}$ and $p_{domestic}$ to be negative. This generally follows from the purchasing power parity (PPP) relation.

Furthermore, the coefficients on the unemployment rate of the non-US country appear to fluctuate between positive and negative signs,^29^ but US unemployment appears to be a significant predictor as it mostly differs from zero, indicating that this variable can function as a proxy for the risk premium.^30^ The coefficients regarding interest rates have the same issue regarding their signs as they again often fluctuate between negative and positive values. Also, the size of these coefficients is, in practical terms, quite negligible, which indicates that they do not significantly affect the forecast. Finally, the changing constant term in some cases implies that there may be some underlying (medium or long-term) process in the economy in which the exchange rate depreciates or appreciates systematically, such as financial deregulation. This seems to be the case for CHF/USD, NZD/USD, and SEK/USD.

In summary, the parameter instability suggests that there is no stable cointegration relation in the data. However, these variables can still have forecasting power in certain periods. This may be because they act as ‘scapegoats’ and/or serve as proxies for changing risk premiums. The difficulty of selecting the best predictive model can be due to frequent shifts in the set of fundamentals driving exchange rates, reflecting swings in market expectations over time [52].

It is important to note that, given the use of an overlapping data and the potential nonstationarity of many explanatory variables, the results must be interpreted with caution. Their reliability hinges on either the stationarity of the explanatory variables or the presence of cointegration. Failure to meet these conditions, combined with the large overlapping period, can induce positive autocorrelations in both the dependent and explanatory variables, potentially leading to spurious regression issues [38], [37]. To assess the robustness of our findings, we examine the stationarity of residuals. Table B.1 presents the results of the Augmented Dickey-Fuller (ADF) test applied to residuals, reporting the median p-values from all estimated DMA models in the out-of-sample exercise. The results indicate that most residuals can be considered stationary at the five percent significance level, which we find promising given the use of overlapping differences that introduce autocorrelation up to order 36. Additionally, we employed the Kwiatkowski-Phillips-Schmidt-Shin (KPSS) test as a further robustness check. The median p-values from all the estimated models are reported in Table B.2. In contrast to the ADF results, the KPSS test yields more mixed results, with residuals generally failing to be considered stationary. On the other hand, it should be noted that DMA in our approach estimates all possible models,^31^ even the ones that make no sense from a theoretical perspective.

Finally, to further support our result regarding significant mean reversion in exchange rates. We estimate the DMA model using only a subset of explanatory variables. More specifically, the only included explanatory variables are the lagged nominal exchange rate, the constant term, and price levels. The price levels are included to account for possible mean reversion in real exchange rates [36].

The results of this DMA model with the subset of explanatory variables are shown in Table 3. The table has the same structure as before and is again divided into sections for each nominal currency pair. The main result is that the model has similar forecasting power in the short run, but in the medium and especially in the long run, it provides significantly worse results for most of the currencies than the full DMA model using all of the variables. In other words, the smaller model has somewhat worse forecasting power. This contrasts with the fact that the posterior inclusion probabilities suggest that the nominal exchange rate forecast could be done with only the lagged value of the nominal exchange rate as an explanatory variable (along with the constant term). Given these results and also taking into account the instability of parameters regarding fundamental variables, all of this suggests some validity of the ‘scapegoat theory of exchange rates, which offers a reconciliation of these findings. The parameters are generally unstable, and the preferred model often changes. However, these variables still have forecasting power in certain periods. The results, therefore, indicate that the inclusion of additional fundamental variables can further exchange rate predictions, which may act as a ‘scapegoat’. The DMA approach is then valuable for its ability to adjust to changes in the fundamentals driving exchange rates, accommodating the dynamic nature of the market.

**Table 3:** Out of sample forecast evaluation - different DMA specification.

AUD/USD	M1	M3	M6	M9	M12	M24	M36
RMSFE_M/RMSFE_RW	1.01	1.00	0.99	0.98	0.89	0.80	0.77
MAFE_M/MAFE_RW	1.00	0.99	0.98	0.98	0.93	0.90	0.90
DMW	0.70	0.49	0.38	0.11	0.09	0.09	0.00
CW	0.26	0.05	0.01	0.00	0.00	0.01	0.00
N	239	237	234	231	228	216	204

**CAD/USD**	M1	M3	M6	M9	M12	M24	M36

RMSFE_M/RMSFE_RW	1.01	1.00	0.98	0.93	0.92	0.77	0.66
MAFE_M/MAFE_RW	1.00	1.01	0.98	0.97	0.94	0.88	0.82
DMW	0.83	0.54	0.35	0.02	0.01	0.07	0.02
CW	0.61	0.09	0.02	0.00	0.00	0.00	0.00
N	239	237	234	231	228	216	204

**CHF/USD**	M1	M3	M6	M9	M12	M24	M36

RMSFE_M/RMSFE_RW	1.01	1.04	0.99	0.97	0.95	0.76	0.54
MAFE_M/MAFE_RW	1.00	1.02	0.99	0.99	0.98	0.86	0.75
DMW	0.93	0.83	0.37	0.25	0.16	0.11	0.14
CW	0.89	0.73	0.11	0.04	0.03	0.01	0.04
N	179	177	174	171	168	156	144

**EUR/USD**	M1	M3	M6	M9	M12	M24	M36

RMSFE_M/RMSFE_RW	1.01	1.00	0.96	0.95	0.97	0.89	0.78
MAFE_M/MAFE_RW	1.00	0.99	0.96	0.98	1.00	0.94	0.90
DMW	0.72	0.50	0.10	0.16	0.30	0.16	0.20
CW	0.48	0.18	0.01	0.00	0.01	0.06	0.08
N	167	165	162	159	156	144	132

**GBP/USD**	M1	M3	M6	M9	M12	M24	M36

RMSFE_M/RMSFE_RW	1.01	1.00	0.98	0.96	0.92	0.62	0.72
MAFE_M/MAFE_RW	1.00	1.00	0.99	0.98	0.96	0.78	0.87
DMW	0.98	0.74	0.10	0.05	0.02	0.04	0.04
CW	0.97	0.48	0.02	0.01	0.00	0.01	0.01
CW	0.81	0.06	0.00	0.00	0.00	0.01	0.01
N	231	229	226	223	220	208	196

**JPY/USD**	M1	M3	M6	M9	M12	M24	M36

RMSFE_M/RMSFE_RW	1.02	1.03	0.98	0.92	0.83	0.62	0.51
MAFE_M/MAFE_RW	1.01	1.02	0.99	0.95	0.90	0.75	0.70
DMW	0.97	0.91	0.25	0.03	0.00	0.01	0.01
CW	0.87	0.57	0.01	0.00	0.00	0.00	0.00
N	194	192	189	186	183	171	159

**NOK/USD**	M1	M3	M6	M9	M12	M24	M36

RMSFE_M/RMSFE_RW	1.01	1.03	1.02	0.97	0.94	0.79	0.69
MAFE_M/MAFE_RW	1.01	1.02	0.99	0.97	0.94	0.91	0.83
DMW	0.99	0.87	0.64	0.23	0.13	0.11	0.12
CW	0.97	0.57	0.25	0.02	0.00	0.00	0.00
N	239	237	234	231	228	216	204

**NZD/USD**	M1	M3	M6	M9	M12	M24	M36

RMSFE_M/RMSFE_RW	1.00	1.01	1.01	1.00	1.01	1.10	1.06
MAFE_M/MAFE_RW	1.00	1.00	1.01	1.01	0.99	1.02	1.01
DMW	0.81	0.68	0.69	0.48	0.55	0.97	0.66
CW	0.64	0.20	0.06	0.03	0.02	0.00	0.27
N	222	220	217	214	211	199	187

**SEK/USD**	M1	M3	M6	M9	M12	M24	M36

RMSFE_M/RMSFE_RW	1.01	1.01	0.98	0.96	0.91	0.80	0.67
MAFE_M/MAFE_RW	1.00	1.00	0.99	0.97	0.95	0.89	0.84
DMW	0.85	0.60	0.29	0.19	0.08	0.13	0.06
CW	0.70	0.22	0.01	0.00	0.00	0.00	0.00
N	180	178	175	172	169	150	140

***Note:*** The dependent variable is the h-step forward difference of the given currency pair. The individual columns (Mx) represent the results for the x month ahead forecast. RMSFE_M/RMSFE_RW is the ratio of root mean squared forecast error of the DMA framework and random walk benchmark. MAFE_M/MAFE_RW represents the ratio of the mean absolute forecast error between the two. DMW and CW indicate the p-values for the Diebold-Mariano and Clark-West test statistics, respectively. N refers to the number of estimated forecasts. DMA framework was used for the estimation and we used only subset of variables.

## 6 Robustness check

In this section, we further build upon our results presented in the previous section that exchange rates can be considered mean-reverting and lagged exchange rates have significant forecasting power. This line of reasoning generally aligns with research by [36], [3], where authors present similar conclusions. We thus further test this hypothesis by comparing the forecasts from the DMA model to benchmarks other than the random walk without drift.

The alternative benchmark model uses a simple direct multi-step forecast (local projection equation) in which the change in the nominal exchange rate from *t* to t+h is a function of a constant and the nominal exchange rate at time *t*. Therefore, an *h* period-ahead forecast is again generated from a different model for every forecasting horizon. The alternative benchmark model is primarily estimated using a standard OLS method. Therefore, the results indicate to what extent additional fundamentals other than the exchange rate help orecast exchange rates. In other words, this allows us to assess if the DMA approach delivers value-added compared to the above simple model. For comparability, we once more use the first 72 periods as the training period. The forecast horizon is again the same as above (one month up to 36 months). However, we use a rolling window estimation approach, where, for each period, the actual realizations of the time series are added as an additional data point at the end of the sample, while the earliest observations are removed. This ensures that the model's training period remains fixed in length throughout the estimation process.^32^ More specifically, we estimate the following model for every currency (see Equation (10)):

(10) $$\Delta_{t+h}e_{i,t}=\alpha_{i,t}+\beta_{i}e_{i,t}+\varepsilon_{i,t+h}$$

where $\alpha_{i}$ is the constant term, and $\beta_{i}$ denotes the slope coefficient. Other notation is the same as in the DMA case. Also, note that for this alternative benchmark model, we do not assume any other explanatory variable except the nominal exchange rate. Furthermore, unlike in the DMA case, the parameters are not considered to be time-varying.^33^

The results of the baseline robustness check are shown in Table 4. The table has the same structure as in the previous section and is again divided into sections for each nominal currency pair. The main results are as follows: The DMA approach generally delivers forecasts beating the OLS benchmark according to RMSFE and MAFE ratios in the medium and long run.^34^ This is especially true in the long run, where the results are even, in most cases, confirmed by DMW statistics. However, in the short-term horizon (up to 6 months), the results of the DMA method relative to the alternative benchmark model are generally comparable. The analysis supports the conclusion that additional fundamentals other than the exchange rate do not help predict exchange rates in the short term from an out-of-sample forecasting perspective. In the medium-term horizon, the DMA contribution to forecasting power is generally modest. However, the DMA method has significantly more forecasting power in the long run than this alternative OLS benchmark, suggesting that additional variables have some forecasting power in these periods. In other words, this suggests that in the long run, fundamental factors have an effect on the exchange rate. From another point of view, the results also suggest that the alternative benchmark results are similar to the random walk benchmark regarding the short-term forecasting horizon. Furthermore, in the medium and long-term horizon, the simple OLS benchmark defeats the random walk benchmark in all cases.^35^ Furthermore, we believe that this supplementary analysis further fosters our argument that direct forecasting greatly helps ensure predictability. In other words, we argue that for an exchange rate forecast capable of defeating a random walk benchmark in the short and medium run is sufficient to use lagged exchange rate values and also the use of a direct forecasting approach, which allows filtering out the non-fundamental noise in the short-term horizon.

**Table 4:** Out of sample forecast evaluation - different benchmark model 1.

AUD/USD	M1	M3	M6	M9	M12	M24	M36
RMSFE_M/RMSFE_B	0.99	0.97	0.89	0.83	0.81	0.64	0.59
MAFE_M/MAFE_B	0.98	0.96	0.90	0.88	0.86	0.79	0.73
DMW	0.35	0.33	0.17	0.14	0.16	0.02	0.02
CW	0.00	0.00	0.00	0.00	0.00	0.00	0.01
N	239	237	234	231	228	216	204

**CAD/USD**	M1	M3	M6	M9	M12	M24	M36

RMSFE_M/RMSFE_B	0.99	0.98	0.94	0.90	0.88	0.75	0.76
MAFE_M/MAFE_B	0.99	0.99	0.96	0.94	0.92	0.90	0.89
DMW	0.27	0.28	0.24	0.17	0.20	0.14	0.17
CW	0.00	0.00	0.00	0.00	0.00	0.01	0.04
N	239	237	234	231	228	216	204

**CHF/USD**	M1	M3	M6	M9	M12	M24	M36

RMSFE_M/RMSFE_B	1.01	1.09	1.07	0.95	0.82	0.77	0.79
MAFE_M/MAFE_B	1.00	1.03	1.00	0.95	0.90	0.89	0.84
DMW	0.74	0.97	0.71	0.35	0.10	0.12	0.12
CW	0.09	0.23	0.05	0.00	0.00	0.01	0.00
N	179	177	174	171	168	156	144

**EUR/USD**	M1	M3	M6	M9	M12	M24	M36

RMSFE_M/RMSFE_B	1.01	1.02	0.86	0.85	0.78	0.75	0.87
MAFE_M/MAFE_B	1.00	1.02	0.93	0.91	0.89	0.87	0.89
DMW	0.65	0.58	0.15	0.22	0.17	0.12	0.05
CW	0.15	0.02	0.01	0.01	0.01	0.03	0.04
N	167	165	162	159	156	144	132

**GBP/USD**	M1	M3	M6	M9	M12	M24	M36

RMSFE_M/RMSFE_B	0.99	1.00	0.93	0.83	0.76	0.65	0.62
MAFE_M/MAFE_B	1.00	1.01	0.97	0.92	0.88	0.79	0.78
DMW	0.35	0.49	0.17	0.07	0.05	0.01	0.05
CW	0.02	0.02	0.00	0.00	0.00	0.00	0.00
N	231	229	226	223	220	208	196

**JPY/USD**	M1	M3	M6	M9	M12	M24	M36

RMSFE_M/RMSFE_B	0.98	0.95	0.84	0.74	0.69	0.54	0.49
MAFE_M/MAFE_B	0.99	0.99	0.93	0.85	0.82	0.73	0.67
DMW	0.22	0.21	0.04	0.03	0.02	0.03	0.01
CW	0.00	0.00	0.00	0.00	0.00	0.00	0.00
N	194	192	189	186	183	171	159

**NOK/USD**	M1	M3	M6	M9	M12	M24	M36

RMSFE_M/RMSFE_B	1.01	1.04	1.00	0.94	0.91	0.77	0.76
MAFE_M/MAFE_B	1.01	1.01	0.98	0.95	0.96	0.91	0.88
DMW	0.64	0.72	0.50	0.35	0.33	0.10	0.12
CW	0.08	0.07	0.01	0.02	0.01	0.01	0.01
N	239	237	234	231	228	216.00	204

**NZD/USD**	M1	M3	M6	M9	M12	M24	M36

RMSFE_M/RMSFE_B	1.01	1.03	1.05	1.03	0.96	0.92	0.89
MAFE_M/MAFE_B	1.01	1.01	1.00	0.98	0.95	0.88	0.91
DMW	0.64	0.65	0.68	0.57	0.38	0.22	0.07
CW	0.06	0.01	0.00	0.00	0.00	0.00	0.00
N	222	220	217	214	211	199	187

**SEK/USD**	M1	M3	M6	M9	M12	M24	M36

RMSFE_M/RMSFE_B	1.02	1.06	1.01	1.02	0.96	0.79	0.78
MAFE_M/MAFE_B	1.01	1.02	0.99	0.98	0.95	0.87	0.86
DMW	0.73	0.75	0.53	0.56	0.39	0.20	0.14
CW	0.12	0.09	0.02	0.02	0.01	0.03	0.04
N	180	178	175	172	169	150	140

***Note:*** The dependent variable is the h-step forward difference of the given currency pair. The individual columns (Mx) represent the results for the x month ahead forecast. RMSFE_M/RMSFE_B is the ratio of root mean squared forecast error of the DMA framework and OLS benchmark. MAFE_M/MAFE_B represents the ratio of the mean absolute forecast error between the two. DMW and CW indicate the p-values for the Diebold-Mariano and Clark-West test statistics, respectively. N refers to the number of estimated forecasts. DMA framework was used for the estimation. A rolling window approach was used for the estimation of the benchmark model.

We also experimented with imposing the constant term to a zero value, i.e., $\alpha_{i}=0$. However, the results of this alternative benchmark model are significantly worse than the DMA approach, as is displayed in Table B.3 in the Appendix, indicating the importance of keeping a constant term that captures the changes in mean caused by other factors or which captures some long-term trend. More importantly, the results are even worse than the random walk benchmark in the long run, further reinforcing the importance of including a constant.

Furthermore, we also used the panel data approach (fixed effects regression) as another benchmark model, i.e., $\Delta_{t+h}e_{i,t}=\alpha_{i,t}+\beta e_{i,t}+\varepsilon_{i,t+h}$. Note that in this case, *β* is without currency index *i*. The rationale behind this approach is that the panel data method may help control estimation errors. The results are shown in Table B.4 in the Appendix. The main findings are as follows: The results are comparable to the DMA methodology only in the case of AUD/USD, GBP/USD, and JPY/USD. On the other hand, regarding the rest of the currencies, the results are significantly worse. In these cases, they are even only significantly worse than the simple OLS benchmark or the random walk benchmark. This suggests different rates of mean reversion regarding different currencies, which the inclusion of different regression terms $\beta_{i}$ for every currency allows us to capture, i.e., separate regression for every currency.

Moreover, for an additional robustness check, we replace the explanatory variable nominal exchange rate in the benchmark model with the real exchange rate. This can be written as follows see Equation (11)):

(11) $$\Delta_{t+h}e_{i,t}=\alpha_{i,t}+\beta_{i}q_{i,t}+\varepsilon_{i,t+h}$$

where $q_{i,t}$ denote the real spot exchange rate. The results of this model are displayed in Table B.5 in the Appendix. It can be said that the results are generally very similar to the benchmark with a nominal exchange rate. In some cases, the results are slightly better, and in some cases, and in others, somewhat worse, but the differences can be considered negligible. This suggests that differences in price levels do not have significant additional forecasting power in the period and countries examined in the simple regression framework.^36^ Again, the argument is that the period under consideration is characterized by mostly low inflation rates and inflation targeting. Therefore, the results suggest that with explicitly set inflation targets, nominal exchange rates and relative price indices can be considered mean reverting.

Finally, we also again experimented with the panel data approach (fixed effects regression) to control for the estimation error. We show the results in Table B.6 in the Appendix. The results are roughly similar to the DMA methodology only in the case of AUD/USD, EUR/USD, and JPY/USD. In other cases, they are once more significantly worse than the DMA approach. Also, the simple OLS benchmark using either nominal or real exchange rate as the explanatory variable generally provides significantly better results. This suggests a different rate of mean reversion even after controlling for the difference in the price levels.

In the robustness check, we, therefore, used five distinct specifications regarding alternative benchmark models other than the random walk benchmark. Overall, only the simple OLS regression benchmark with the real or nominal exchange rate as the explanatory variable, unrestricted constant term, and estimated for each currency separately turns out to be consistently better than the random walk benchmark. Furthermore, short-term forecasts are broadly comparable to the predictions from the DMA model. However, the DMA framework generally produces somewhat better results in the medium run and significantly better results in the long run.

## 7 Conclusion

This paper presents a dynamic model averaging (DMA) approach for forecasting exchange rates. The main contribution of this article is the use of this method, which is relatively novel in exchange rate forecasting literature and allows us to study parameters and model uncertainty in exchange rate forecasting. Specifically, the method allows us to assess many different model specifications and posterior inclusion probabilities of all the variables considered.

The study empirically shows the medium and long-term predictability of nine liquid currencies (AUD/USD, CAD/USD, CHF/USD, EUR/USD, GBP/USD, JPY/USD, NOK/USD, NZD/USD, and SEK/USD) in approximately the last two decades using monthly data. In the out-of-sample forecasting exercise, we use a direct multi-step forecast combined with a dynamic model averaging framework. This approach delivers a forecast that defeats the random walk without drift benchmark based on the RMSFE and MAFE ratios since the nine-month ahead forecast period for all currencies. Overall, the study presents substantive findings regarding statistically and economically significant exchange rate predictability in the medium and long run for all nine currency pairs. We also present some findings on predictability and forecasting performance, even for the short run, although in this case, the results are much less impressive.

Specifically, the long-term forecasts—namely, those for two and three years ahead—appear to be particularly impressive. The model outperforms the random walk without drift, even when the specification is altered to include only the lagged nominal exchange rate, the constant, and price levels. Our results show that this simplified model, however, performs slightly worse in the medium and long term for most of the currency pairs examined. Our results suggest that, at a minimum, studies making medium and long-term exchange rate forecasts should include lagged nominal exchange rates, a constant term, and price levels as explanatory variables. The results further indicate that the inclusion of additional variables can improve exchange rate predictions. However, the parameter values are generally unstable and country-dependent. Despite this variability, these variables can still possess forecasting power during certain periods, which is accommodated by the DMA framework.

On the contrary, the short-term predictive power is not particularly strong; however, the results are comparable to the random walk benchmark when evaluated using the RMSFE and MAFE ratios. However, our approach still defeats random walk in eight out of the nine cases in six months ahead forecast. In short, in forecasting for very short-term periods, typically one to three months, standard exchange rate models, even in the DMA framework, are not particularly successful. Therefore, the random walk model likely remains the best benchmark in these scenarios. For these periods, future research may need to use a completely different methodology or focus on variables other than ‘fundamental’ factors, as these variables may not sufficiently capture the short-term drivers of exchange rates. For longer periods, the DMA framework can yield better results, although outcomes can vary depending on the currency.

In general, the model's performance improves as the forecasting horizon extends, although the results may still be affected by the typical limitations associated with long-horizon forecasts. In more detail, [3] highlight that the results of many prediction models may be exaggerated due to a lack of awareness of small sample bias, which is a significant drawback in long-horizon forecasts. When time series are highly persistent, a sample size of 20 years may be considered relatively small, leading to test statistics that are subject to considerable biases not easily corrected with standard statistical methods. Additionally, as the literature has shown, it is also challenging to assess this small sample bias using simulation methods. This issue is even more pronounced within the DMA framework, as we are not aware of any study that has attempted to address this bias using simulation methods within this framework.

In addition, we also present several possible explanations for our results regarding exchange rate predictability. One potential explanation is that by using a large number of variables, we implicitly identified the cointegration vector, which means that there exists some fundamental value to which the nominal exchange rate converges. The long-run forecasts suggest some validity of this theory. On the other hand, the instability of many parameters considered in the DMA framework challenges this explanation. The possible reconciliation of these findings can be offered by the ‘scapegoat theory of exchange rates’. However, based on the short and medium-term results, we also claim that there exists significant mean reversion in nominal exchange rates, at least in the period examined, i.e., approximately the last two decades. This is in line with [36] view of stationary real exchange rates. In a mostly low inflation environment and with central banks following explicitly set inflation targets, it makes sense that the nominal rate is also stationary. Also, note that the ‘scapegoat theory of exchange rates’ and the mean reversion in exchange rates are not contradictory. The exchange rate may be stationary, but other variables may help to predict its value.

Furthermore, the use of a direct multi-step forecast is another important modelling decision regarding significant exchange rate predictability in the medium and long run. This is because exchange rate dynamics are often obscured by high-frequency ‘non-fundamental’ noise, which is inherently difficult to predict. By using this forecasting approach, we were able to filter out this noise. In other words, the prediction errors do not accumulate over time due to the use of direct forecasts. This interpretation is further strengthened in the robustness check section, where we used several different benchmark models. Most successful alternative benchmark models use a simple direct multi-step OLS forecast in which the change in the exchange rate is a function of a constant and the lagged level of the nominal or real exchange rate. These models achieve similar results in the short and medium term, but the DMA approach in the long-term horizon defeats them. These findings again suggest that, in the long run, fundamental factors have some effect on the exchange rate, but their impact is relatively modest in the short and medium term.

## CRediT authorship contribution statement

**Martin Časta:** Writing – review & editing, Writing – original draft, Visualization, Validation, Supervision, Software, Resources, Project administration, Methodology, Investigation, Funding acquisition, Formal analysis, Data curation, Conceptualization.

## Declaration of Competing Interest

The authors declare that they have no known competing financial interests or personal relationships that could have appeared to influence the work reported in this paper.

## Figures and Tables

**Table A.1:** Data description.

Name (abbreviated)	Ticker	Source	Country
U.S. Dollars to Australian Dollar Spot Exchange Rate	DEXUSAL	FRED	AUD/USD
Canadian Dollars to U.S. Dollar Spot Exchange Rate	DEXCAUS	FRED	CAD/USD
Swiss Francs to US Dollar Spot Exchange Rate	DEXSZUS	FRED	CHF/USD
U.S. Dollars to Euro Spot Exchange Rate	DEXUSEU	FRED	EUR/USD
U.S. Dollars to U.K. Pound Sterling Spot Exchange Rate	DEXUSUK	FRED	GBP/USD
Japanese Yen to U.S. Dollar Spot Exchange Rate	DEXJPUS	FRED	JPY/USD
Norwegian Kroner to U.S. Dollar Spot Exchange Rate	DEXNOUS	FRED	NOK/USD
U.S. Dollars to New Zealand Dollar Spot Exchange Rate	DEXUSNZ	FRED	NZD/USD
Swedish Kronor to U.S. Dollar Spot Exchange Rate	DEXSDUS	FRED	SWD/USD

Credit; Business Banks	D2 LENDING AND CREDIT AGGREGATES	RBA	AU
Credit liabilities of private non-financial corporations		BoC	CA
Domestic loans (utilisation and credit lines)	EPB@SNB.bakredbetgrbmAV1,KC5A,T1,F	SNB	CH
Domestic loans (utilisation and credit lines)	EPB@SNB.bakredbetgrbmAV1,KC5A,T1,B	SNB	CH
Domestic loans (utilisation and credit lines)	EPB@SNB.bakredbetgrbmAV1,KC5B,T1,F	SNB	CH
Domestic loans (utilisation and credit lines)	EPB@SNB.bakredbetgrbmAV1,KC5B,T1,B	SNB	CH
Domestic loans (utilisation and credit lines)	EPB@SNB.bakredbetgrbmAV1,KC5C,T1,F	SNB	CH
Domestic loans (utilisation and credit lines)	EPB@SNB.bakredbetgrbmAV1,KC5C,T1,B	SNB	CH
Domestic loans (utilisation and credit lines)	EPB@SNB.bakredbetgrbmAV1,KC5D,T1,F	SNB	CH
Domestic loans (utilisation and credit lines)	EPB@SNB.bakredbetgrbmAV1,KC5D,T1,B	SNB	CH
Loans vis-a-vis euro area NFC reported by MFI	BSI.M.U2.N.A.A20.A.1.U2.2240.Z01.E	ECB	EU
Outstanding Loans and Bills Discounted/Corporations	MD11'DLCLAEDBLTCO	BoJ	JAP
Total Credit to Non-Financial Corporations	CRDQNOANUBIS	FRED	NO
Sector lending	CRDS.MALP3	RBNZ	NZL
Lending, Swe, Non-financial corporations		SCB	SWE
UK M4 LENDING: PRIVATE NON-FINANCIAL	UKBANKLPB	Datastream	UK
Commercial and Industrial Loans, All Commercial Banks	BUSLOANSNSA	FRED	US

Consumer Price Index of All Items in Australia	AUSCPIALLQINMEI	FRED	AU
Consumer Price Index for All Urban Consumers	CPIAUCSL	FRED	CA
Consumer Price Index: All Items for Switzerland	CHECPIALLMINMEI	FRED	CH
HICP - monthly data (index)	PRC_HICP_MIDX	Eurostat	EU
Consumer Price Index of All Items in Japan	JPNCPIALLMINMEI	FRED	JAP
Consumer Price Index: All Items for Norway	NORCPIALLMINMEI	FRED	NO
Consumer Price Index: All Items for New Zealand	NZLCPIALLQINMEI	FRED	NZL
Consumer Price Index: All Items for Sweden	SWECPIALLMINMEI	FRED	SWE
Consumer Price Index of All Items in the United Kingdom	GBRCPIALLMINMEI	FRED	UK
Consumer Price Index for All Urban Consumers	CPIAUCSL	FRED	US

Harmonized Unemployment Rate: Australia	LRHUTTTTAUM156N	FRED	AU
Unemployment Rate: Aged 15 and Over: Canada	LRUNTTTTCAM156N	FRED	CA
Unemployment by sex and age	UNE_RT_M	Eurostat	CH
Unemployment by sex and age	UNE_RT_M	Eurostat	EU
Unemployment Rate: Aged 15-64: All Persons for Japan	LRUN64TTJPM156N	FRED	JAP
Registered Unemployment Level for Norway	LMUNRRTTNOM156N	FRED	NO
Registered Unemployment Level for New Zealand	LMUNRLTTNZM647N	FRED	NZL
Unemployment Rate: New Zealand	LRUNTTTTNZQ156S	FRED	NZL
Harmonized Unemployment Rate: Total: Sweden	UNRATENSA	FRED	SWE
Harmonized Unemployment Rate: United Kingdom	LRHUTTTTGBM156N	FRED	UK
Unemployment Rate, Percent, Monthly	UNRATENSA	FRED	US

Interest Rates: 3-Month or 90-Day Rates and Yields: Australia	IR3TIB01AUM156N	FRED	AU
Interest Rates: 3-Month or 90-Day Rates and Yields: Canada	IR3TIB01CAM156N	FRED	CA
Interest Rates: 3-Month or 90-Day Rates and Yields: Switzerland	IR3TIB01CHM156N	FRED	CH
Interest Rates: 3-Month or 90-Day Rates and Yields: Euro Area	IR3TIB01EZM156N	FRED	EU
Japanese Yen TIBOR	3MONTH	jbatibor	JAP
3-Month or 90-day Rates and Yields: Norway	IR3TIB01NOM156N	FRED	NO
3-Month or 90-day Rates and Yields: New Zealand	IR3TIB01NZM156N	FRED	NZL
3-Month or 90-day Rates and Yields: Sweden	IR3TIB01SEM156N	FRED	SWE
3-Month or 90-day Rates and Yields: United Kingdom	IR3TIB01GBM156N	FRED	UK
3-Month or 90-day Rates and Yields: United States	IR3TIB01USM156N	FRED	US

**Table A.2:** Summary statistics.

Variable	Mean	Sd	Max	Min	Rho	ADF	KPSS	N
**Nominal Exchange Rate**								

AUD/USD	-0.29	0.18	0.10	-0.72	0.98	0.56	0.01	312
CAD/USD	-0.22	0.14	0.05	-0.47	0.98	0.85	0.01	312
CHF/USD	-0.05	0.15	0.24	-0.54	0.96	0.34	0.01	252
EUR/USD	0.21	0.10	0.46	-0.02	0.96	0.03	0.01	240
GBP/USD	0.44	0.13	0.73	0.11	0.98	0.42	0.01	304
JPY/USD	-0.05	0.15	0.24	-0.54	0.96	0.34	0.01	252
NZD_USD	-0.43	0.19	-0.13	-0.93	0.98	0.56	0.01	295
SEK_USD	-2.06	0.15	-1.78	-2.41	0.96	0.17	0.01	253

**Credit to Corporation (Money)**								

Australia	6.38	0.44	6.96	5.47	0.99	0.74	0.01	312
Canada	6.74	0.44	7.58	5.98	0.99	0.84	0.01	312
Switzerland	13.48	0.21	13.91	13.16	0.99	0.20	0.01	252
Euro Area	8.35	0.15	8.55	8.00	0.98	0.41	0.01	240
Great Britain	5.80	0.31	6.21	5.12	0.99	0.75	0.01	304
Japan	14.91	0.10	15.14	14.79	0.98	0.36	0.01	267
New Zealand	11.22	0.38	11.80	10.46	0.99	0.69	0.01	295
Sweden	14.30	0.28	14.91	13.84	0.98	0.91	0.01	253
United States	7.25	0.37	8.03	6.66	0.99	0.44	0.01	312

**Price level**								

Australia	4.46	0.19	4.81	4.12	0.99	0.59	0.01	312
Canada	4.51	0.13	4.80	4.28	0.99	0.96	0.01	312
Switzerland	4.61	0.02	4.65	4.55	0.97	0.39	0.01	252
Euro Area	4.57	0.09	4.80	4.39	0.98	0.53	0.01	240
Great Britain	4.50	0.15	4.83	4.25	0.99	0.75	0.01	304
Japan	4.59	0.02	4.66	4.56	0.97	0.92	0.01	267
New Zealand	4.51	0.16	4.84	4.24	0.99	0.99	0.01	312
Sweden	4.59	0.08	4.84	4.45	0.97	0.99	0.01	253
United States	5.36	0.16	5.70	5.07	0.99	0.92	0.01	312

**Unemployment rate**								

Australia	1.72	0.19	2.27	1.17	0.93	0.50	0.01	312
Canada	1.96	0.17	2.62	1.50	0.84	0.02	0.01	312
Switzerland	1.61	0.36	2.88	0.74	0.91	0.59	0.01	252
Euro Area	2.21	0.17	2.55	1.86	0.98	0.96	0.01	240
Great Britain	1.72	0.22	2.15	1.28	0.99	0.87	0.01	288
Japan	1.38	0.26	1.79	0.79	0.98	0.52	0.01	267
New Zealand	1.59	0.24	2.07	1.16	0.97	0.56	0.01	295
Sweden	2.00	0.15	2.33	1.67	0.78	0.14	0.03	253
United States	1.69	0.30	2.67	1.19	0.94	0.58	0.02	312

**Interest rate**								

Australia	3.85	2.00	7.90	0.01	0.99	0.09	0.01	312
Canada	2.39	1.64	5.92	0.18	0.99	0.38	0.01	312
Switzerland	0.59	1.24	3.58	-0.86	0.99	0.29	0.01	312
Euro Area	1.71	1.85	5.11	-0.58	0.99	0.33	0.01	312
Great Britain	0.59	1.24	3.58	-0.86	0.99	0.29	0.01	312
Japan	0.22	0.24	0.89	-0.07	0.99	0.70	0.01	276
New Zealand	4.33	2.47	9.26	0.26	0.99	0.20	0.01	312
Sweden	1.65	1.78	4.49	-0.79	0.99	0.43	0.01	312
United States	2.27	2.12	6.73	0.09	0.99	0.55	0.01	312

***Note:*** The table shows the time series which are used in the forecasting exercise. All variables are in log form. Rho denotes the autocorrelation coefficient at first lag. N denotes the number of observations. ADF and KPSS denote p-values of Augmented Dickey-Fuller and Kwiatkowski–Phillips–Schmidt–Shin statistics. The number of lags in ADF and KPSS tests was selected to minimize the AIC.

**Table B.1:** Adf test P-values (median values over all DMA models).

	M1	M3	M6	M9	M12	M24	M36
AUD_USD	0.01	0.01	0.01	0.01	0.01	0.01	0.01
CAD_USD	0.01	0.01	0.01	0.01	0.01	0.01	0.01
CHF_USD	0.01	0.01	0.01	0.01	0.01	0.01	0.01
EUR_USD	0.02	0.01	0.01	0.01	0.01	0.01	0.01
GBP_USD	0.01	0.01	0.01	0.01	0.01	0.01	0.01
JPY_USD	0.01	0.01	0.01	0.01	0.01	0.01	0.01
NOK_USD	0.01	0.01	0.01	0.01	0.01	0.01	0.0
NZD_USD	0.01	0.01	0.01	0.01	0.01	0.01	0.01
SEK_USD	0.01	0.01	0.01	0.01	0.01	0.01	0.01

***Note:*** ADF tests the null hypothesis that a unit root is present. The number of lags was selected to minimize the AIC. P-value 0.01 means equal or smaller than 0.01. The individual columns (Mx) denote the results for the x month ahead forecast.

**Table B.2:** KPSS test P-values (median values over all DMA models).

	M1	M3	M6	M9	M12	M24	M36
AUD_USD	0.01	0.01	0.03	0.02	0.02	0.02	0.01
CAD_USD	0.01	0.01	0.01	0.02	0.01	0.01	0.03
CHF_USD	0.10	0.10	0.01	0.01	0.1	0.10	0.02
EUR_USD	0.01	0.01	0.01	0.01	0.02	0.01	0.01
GBP_USD	0.01	0.02	0.01	0.03	0.01	0.05	0.01
JPY_USD	0.01	0.01	0.02	0.02	0.10	0.02	0.01
NOK_USD	0.05	0.01	0.01	0.01	0.01	0.01	0.01
NZD_USD	0.01	0.01	0.01	0.02	0.01	0.01	0.10
SEK_USD	0.01	0.01	0.01	0.02	0.01	0.01	0.01

***Note:*** KPSS tests the null hypothesis that time series are stationary. The number of lags was selected to minimize the AIC. P-value 0.10 means equal or greater than 0.10. The individual columns denote the results for the x month ahead forecast.

**Table B.3:** Out of sample forecast evaluation - different benchmark model 2.

AUD/USD	M1	M3	M6	M9	M12	M24	M36
RMSFE_M/RMSFE_B	1.01	0.99	0.93	0.86	0.82	0.61	0.50
MAFE_M/MAFE_B	1.00	0.98	0.94	0.90	0.87	0.77	0.67
DMW	0.73	0.45	0.03	0.02	0.02	0.00	0.00
CW	0.04	0.00	0.00	0.00	0.00	0.00	0.00
N	239	237	234	231	228	216	204

**CAD/USD**	M1	M3	M6	M9	M12	M24	M36

RMSFE_M/RMSFE_B	1.00	0.97	0.94	0.87	0.84	0.70	0.65
MAFE_M/MAFE_B	1.00	0.98	0.96	0.92	0.88	0.83	0.80
DMW	0.59	0.19	0.12	0.01	0.01	0.02	0.00
CW	0.01	0.00	0.00	0.00	0.00	0.00	0.00
N	239	237	234	231	228	216	204

**CHF/USD**	M1	M3	M6	M9	M12	M24	M36

RMSFE_M/RMSFE_B	1.04	1.10	1.06	0.92	0.77	0.56	0.47
MAFE_M/MAFE_B	1.01	1.04	1.00	0.95	0.89	0.76	0.68
DMW	0.97	0.99	0.78	0.20	0.06	0.07	0.13
CW	0.89	0.94	0.19	0.01	0.01	0.01	0.03
N	179	177	174	171	168	156	144

**EUR/USD**	M1	M3	M6	M9	M12	M24	M36

RMSFE_M/RMSFE_B	0.95	0.86	0.62	0.55	0.47	0.43	0.55
MAFE_M/MAFE_B	0.97	0.94	0.80	0.73	0.69	0.64	0.70
DMW	0.06	0.07	0.02	0.02	0.01	0.01	0.11
CW	0.00	0.00	0.00	0.00	0.00	0.00	0.01
N	167	165	162	159	156	144	132

**GBP/USD**	M1	M3	M6	M9	M12	M24	M36

RMSFE_M/RMSFE_B	0.78	0.62	0.45	0.36	0.32	0.21	0.20
MAFE_M/MAFE_B	0.89	0.80	0.68	0.62	0.58	0.46	0.44
DMW	0.00	0.00	0.01	0.01	0.02	0.03	0.02
CW	0.00	0.00	0.00	0.00	0.00	0.01	0.00
N	231	229	226	223	220	208	196

**JPY/USD**	M1	M3	M6	M9	M12	M24	M36

RMSFE_M/RMSFE_B	0.02	0.01	0.01	0.01	0.01	0.00	0.00
MAFE_M/MAFE_B	0.13	0.10	0.08	0.07	0.07	0.06	0.05
DMW	0.00	0.00	0.00	0.00	0.00	0.00	0.00
CW	0.00	0.00	0.00	0.00	0.00	0.00	0.00
N	194	192	189	186	183	171	159

**NOK/USD**	M1	M3	M6	M9	M12	M24	M36

RMSFE_M/RMSFE_B	0.11	0.06	0.05	0.04	0.03	0.03	0.03
MAFE_M/MAFE_B	0.33	0.24	0.20	0.18	0.18	0.17	0.16
DMW	0.00	0.00	0.00	0.00	0.00	0.00	0.00
CW	0.00	0.00	0.00	0.00	0.00	0.00	0.00
N	239	237	234	231	228	216.00	204

**NZD/USD**	M1	M3	M6	M9	M12	M24	M36

RMSFE_M/RMSFE_B	0.97	0.91	0.87	0.79	0.71	0.64	0.63
MAFE_M/MAFE_B	0.97	0.91	0.87	0.79	0.71	0.64	0.63
DMW	0.14	0.13	0.10	0.02	0.02	0.00	0.01
CW	0.00	0.00	0.00	0.00	0.00	0.00	0.00
N	222	220	217	214	211	199	187

**SEK/USD**	M1	M3	M6	M9	M12	M24	M36

RMSFE_M/RMSFE_B	0.10	0.06	0.04	0.03	0.03	0.02	0.02
MAFE_M/MAFE_B	0.29	0.22	0.17	0.15	0.14	0.12	0.14
DMW	0.00	0.00	0.00	0.00	0.00	0.00	0.00
CW	0.00	0.00	0.00	0.00	0.00	0.00	0.00
N	180	178	175	172	169	150	140

***Note:*** The dependent variable is the h-step forward difference of the given currency pair. The individual columns (Mx) represent the results for the x month ahead forecast. RMSFE_M/RMSFE_B denote the ratio of root mean squared forecast error of the DMA framework and OLS benchmark. MAFE_M/MAFE_B represents the ratio of the mean absolute forecast error between the two. DMW and CW indicate the p-values for the Diebold-Mariano and Clark-West test statistics, respectively. N refers to the number of estimated forecasts. DMA framework was used for the estimation. A rolling window approach was used for the estimation of the benchmark model.

**Table B.4:** Out of sample forecast evaluation - different benchmark model 3.

AUD/USD	M1	M3	M6	M9	M12	M24	M36
RMSFE_M/RMSFE_B	1.00	1.07	1.15	1.16	1.11	1.06	1.16
MAFE_M/MAFE_B	1.01	1.04	1.07	1.06	1.04	0.95	0.99
DMW	0.54	0.82	0.85	0.90	0.82	0.61	0.68
CW	0.00	0.00	0.02	0.04	0.05	0.13	0.27
N	167	165	162	159	156	144	132

**CAD/USD**	M1	M3	M6	M9	M12	M24	M36

RMSFE_M/RMSFE_B	0.24	0.14	0.11	0.09	0.09	0.08	0.07
MAFE_M/MAFE_B	0.42	0.33	0.29	0.26	0.26	0.25	0.24
DMW	0.00	0.00	0.00	0.00	0.00	0.00	0.00
CW	0.00	0.00	0.00	0.00	0.00	0.00	0.00
N	167	165	162	159	156	144	132

**CHF/USD**	M1	M3	M6	M9	M12	M24	M36

RMSFE_M/RMSFE_B	0.98	0.93	0.86	0.82	0.78	0.62	0.50
MAFE_M/MAFE_B	0.99	0.98	0.92	0.89	0.87	0.75	0.67
DMW	0.09	0.04	0.01	0.01	0.00	0.00	0.00
CW	0.00	0.00	0.00	0.00	0.00	0.00	0.00
N	167	165	162	159	156	144	132

**EUR/USD**	M1	M3	M6	M9	M12	M24	M36

RMSFE_M/RMSFE_B	0.94	0.93	0.94	0.87	0.84	0.84	0.67
MAFE_M/MAFE_B	0.97	0.97	0.98	0.92	0.91	0.90	0.81
DMW	0.04	0.21	0.34	0.20	0.19	0.23	0.20
CW	0.00	0.00	0.00	0.00	0.00	0.00	0.00
N	167	165	162	159	156	144	132

**GBP/USD**	M1	M3	M6	M9	M12	M24	M36

RMSFE_M/RMSFE_B	0.98	1.00	1.01	0.98	0.95	0.94	1.10
MAFE_M/MAFE_B	0.99	1.01	0.99	0.99	0.97	0.99	1.03
DMW	0.10	0.50	0.54	0.39	0.32	0.36	0.99
CW	0.03	0.22	0.21	0.07	0.05	0.09	0.21
N	167	165	162	159	156	144	132

**JPY/USD**	M1	M3	M6	M9	M12	M24	M36

RMSFE_M/RMSFE_B	0.97	0.93	0.91	0.91	0.91	0.98	1.26
MAFE_M/MAFE_B	0.98	0.97	0.95	0.95	0.94	1.00	1.14
DMW	0.08	0.06	0.16	0.29	0.31	0.47	0.87
CW	0.00	0.00	0.01	0.04	0.06	0.15	0.42
N	167	165	162	159	156	144	132

**NOK/USD**	M1	M3	M6	M9	M12	M24	M36

RMSFE_M/RMSFE_B	0.98	0.91	0.77	0.68	0.62	0.52	0.46
MAFE_M/MAFE_B	0.98	0.95	0.88	0.81	0.78	0.72	0.69
DMW	0.13	0.07	0.02	0.02	0.03	0.11	0.12
CW	0.00	0.00	0.00	0.00	0.00	0.02	0.01
N	167	165	162	159	156	144	132

**NZD/USD**	M1	M3	M6	M9	M12	M24	M36

RMSFE_M/RMSFE_B	0.67	0.50	0.42	0.38	0.37	0.23	0.19
MAFE_M/MAFE_B	0.78	0.63	0.56	0.53	0.51	0.43	0.38
DMW	0.00	0.00	0.00	0.00	0.00	0.00	0.00
CW	0.00	0.00	0.00	0.00	0.00	0.00	0.00
N	167	165	162	159	156	144	132

**SEK/USD**	M1	M3	M6	M9	M12	M24	M36

RMSFE_M/RMSFE_B	0.58	0.35	0.22	0.18	0.15	0.10	0.09
MAFE_M/MAFE_B	0.73	0.54	0.42	0.38	0.35	0.28	0.26
DMW	0.00	0.00	0.00	0.00	0.00	0.00	0.00
CW	0.00	0.00	0.00	0.00	0.00	0.00	0.00
N	167	165	162	159	156	144	132

***Note:*** The dependent variable is the h-step forward difference of the given currency pair. The individual columns (Mx) represent the results for the x month ahead forecast. RMSFE_M/RMSFE_B is the ratio of root mean squared forecast error of the DMA framework and Panel regression benchmark (fixed effects regression with nominal exchange rate as explanatory variable). MAFE_M/MAFE_B represents the ratio of the mean absolute forecast error between the two. DMW and CW indicate the p-values for the Diebold-Mariano and Clark-West test statistics, respectively. N refers to the number of estimated forecasts. DMA framework was used for the estimation. A rolling window approach was used for the estimation of the benchmark model.

**Table B.5:** Out of sample forecast evaluation - different benchmark model 4.

AUD/USD	M1	M3	M6	M9	M12	M24	M36
RMSFE_M/RMSFE_B	0.99	0.96	0.88	0.82	0.80	0.65	0.60
MAFE_M/MAFE_B	0.98	0.95	0.89	0.88	0.86	0.80	0.74
DMW	0.28	0.28	0.14	0.12	0.15	0.02	0.03
CW	0.00	0.00	0.00	0.00	0.00	0.00	0.01
N	239	237	234	231	228	216	204

**CAD/USD**	M1	M3	M6	M9	M12	M24	M36

RMSFE_M/RMSFE_B	0.99	0.98	0.95	0.90	0.88	0.75	0.75
MAFE_M/MAFE_B	0.99	1.00	0.96	0.94	0.92	0.90	0.90
DMW	0.36	0.35	0.28	0.19	0.22	0.14	0.17
CW	0.01	0.00	0.00	0.00	0.00	0.01	0.04
N	239	237	234	231	228	216	204

**CHF/USD**	M1	M3	M6	M9	M12	M24	M36

RMSFE_M/RMSFE_B	1.01	1.07	1.02	0.88	0.74	0.64	0.65
MAFE_M/MAFE_B	1.00	1.03	0.98	0.91	0.86	0.80	0.76
DMW	0.72	0.89	0.57	0.21	0.08	0.05	0.06
CW	0.11	0.09	0.02	0.00	0.00	0.01	0.00
N	179	177	174	171	168	156	144

**EUR/USD**	M1	M3	M6	M9	M12	M24	M36

RMSFE_M/RMSFE_B	1.00	1.01	0.85	0.81	0.73	0.71	0.85
MAFE_M/MAFE_B	1.00	1.01	0.92	0.90	0.87	0.84	0.88
DMW	0.57	0.54	0.15	0.17	0.13	0.06	0.07
CW	0.12	0.02	0.01	0.01	0.01	0.01	0.03
N	167	165	162	159	156	144	132

**GBP/USD**	M1	M3	M6	M9	M12	M24	M36

RMSFE_M/RMSFE_B	0.99	0.98	0.90	0.81	0.75	0.65	0.64
MAFE_M/MAFE_B	1.00	1.00	0.96	0.92	0.88	0.80	0.79
DMW	0.27	0.36	0.12	0.05	0.05	0.02	0.05
CW	0.02	0.01	0.00	0.00	0.00	0.00	0.00
N	231	229	226	223	220	208	196

**JPY/USD**	M1	M3	M6	M9	M12	M24	M36

RMSFE_M/RMSFE_B	0.99	0.96	0.84	0.74	0.68	0.52	0.47
MAFE_M/MAFE_B	0.99	0.99	0.93	0.85	0.83	0.74	0.68
DMW	0.35	0.23	0.05	0.03	0.03	0.05	0.02
CW	0.01	0.00	0.00	0.00	0.00	0.01	0.01
N	194	192	189	186	183	171	159

**NOK/USD**	M1	M3	M6	M9	M12	M24	M36

RMSFE_M/RMSFE_B	1.00	1.03	1.00	0.94	0.91	0.77	0.76
MAFE_M/MAFE_B	1.00	1.01	0.98	0.94	0.95	0.90	0.88
DMW	0.56	0.69	0.51	0.37	0.33	0.09	0.11
CW	0.04	0.05	0.01	0.01	0.01	0.00	0.01
N	239	237	234	231	228	216	204

**NZD/USD**	M1	M3	M6	M9	M12	M24	M36

RMSFE_M/RMSFE_B	1.00	1.01	1.01	0.98	0.91	0.88	0.87
MAFE_M/MAFE_B	1.00	1.01	0.99	0.95	0.92	0.87	0.90
DMW	0.56	0.58	0.55	0.45	0.27	0.17	0.03
CW	0.04	0.01	0.00	0.00	0.00	0.00	0.00
N	222	220	217	214	211	199	187

**SEK/USD**	M1	M3	M6	M9	M12	M24	M36

RMSFE_M/RMSFE_B	1.01	1.04	0.99	0.98	0.90	0.74	0.74
MAFE_M/MAFE_B	1.00	1.02	1.00	0.97	0.95	0.86	0.86
DMW	0.64	0.67	0.45	0.45	0.30	0.20	0.17
CW	0.09	0.07	0.01	0.02	0.01	0.05	0.05
N	180	178	175	172	169	150	140

***Note:*** The dependent variable is the h-step forward difference of the given currency pair. The individual columns (Mx) represent the results for the x month ahead forecast. RMSFE_M/RMSFE_B is the ratio of root mean squared forecast error of the DMA framework and OLS benchmark (with real exchange rate). MAFE_M/MAFE_B represents the ratio of the mean absolute forecast error between the two. DMW and CW indicate the p-values for the Diebold-Mariano and Clark-West test statistics, respectively. N refers to the number of estimated forecasts. DMA framework was used for the estimation. A rolling window approach was used for the estimation of the benchmark model.

**Table B.6:** Out of sample forecast evaluation - different benchmark model 5.

AUD/USD	M1	M3	M6	M9	M12	M24	M36
RMSFE_M/RMSFE_B	1.02	1.14	1.32	1.46	1.52	2.01	2.24
MAFE_M/MAFE_B	1.02	1.07	1.12	1.16	1.18	1.30	1.36
DMW	0.87	0.98	0.96	0.97	0.97	0.90	0.85
CW	0.18	0.63	0.78	0.70	0.52	0.71	0.64
N	167	165	162	159	156	144	132

**CAD/USD**	M1	M3	M6	M9	M12	M24	M36

RMSFE_M/RMSFE_B	0.62	0.41	0.30	0.26	0.24	0.22	0.19
MAFE_M/MAFE_B	0.73	0.57	0.48	0.44	0.43	0.42	0.38
DMW	0.00	0.00	0.00	0.00	0.00	0.00	0.00
CW	0.00	0.00	0.00	0.00	0.00	0.00	0.00
N	167	165	162	159	156	144	132

**CHF/USD**	M1	M3	M6	M9	M12	M24	M36

RMSFE_M/RMSFE_B	1.00	0.97	0.93	0.92	0.90	0.80	0.68
MAFE_M/MAFE_B	0.99	0.98	0.94	0.93	0.93	0.86	0.81
DMW	0.41	0.19	0.16	0.17	0.16	0.13	0.11
CW	0.02	0.01	0.01	0.00	0.00	0.00	0.00
N	167	165	162	159	156	144	132

**EUR/USD**	M1	M3	M6	M9	M12	M24	M36

RMSFE_M/RMSFE_B	1.01	1.09	1.16	1.16	1.19	1.32	1.27
MAFE_M/MAFE_B	1.02	1.12	1.29	1.35	1.43	1.78	1.67
DMW	0.85	1.00	1.00	1.00	1.00	1.00	0.97
CW	0.33	0.90	0.99	0.89	0.80	0.98	0.69
N	167	165	162	159	156	144	132

**GBP/USD**	M1	M3	M6	M9	M12	M24	M36

RMSFE_M/RMSFE_B	0.99	0.97	0.94	0.93	0.90	0.88	0.80
MAFE_M/MAFE_B	0.97	0.96	0.98	0.95	0.91	0.84	0.80
DMW	0.06	0.20	0.42	0.40	0.35	0.32	0.32
CW	0.00	0.00	0.00	0.00	0.00	0.00	0.01
N	167	165	162	159	156	144	132

**JPY/USD**	M1	M3	M6	M9	M12	M24	M36

RMSFE_M/RMSFE_B	0.99	0.98	1.00	1.05	1.06	1.03	1.13
MAFE_M/MAFE_B	0.99	0.98	1.01	1.02	1.03	1.05	1.12
DMW	0.27	0.35	0.51	0.60	0.59	0.53	0.61
CW	0.00	0.00	0.00	0.01	0.02	0.02	0.00
N	167	165	162	159	156	144	132

**NOK/USD**	M1	M3	M6	M9	M12	M24	M36

RMSFE_M/RMSFE_B	1.02	1.00	0.85	0.72	0.65	0.53	0.40
MAFE_M/MAFE_B	1.01	0.99	0.90	0.83	0.78	0.70	0.61
DMW	0.71	0.49	0.18	0.06	0.02	0.00	0.01
CW	0.15	0.01	0.00	0.00	0.00	0.00	0.00
N	167	165	162	159	156	144	132

**NZD/USD**	M1	M3	M6	M9	M12	M24	M36

RMSFE_M/RMSFE_B	0.88	0.77	0.68	0.62	0.59	0.39	0.30
MAFE_M/MAFE_B	0.92	0.84	0.76	0.70	0.67	0.55	0.48
DMW	0.00	0.00	0.00	0.00	0.00	0.00	0.00
CW	0.00	0.00	0.00	0.00	0.00	0.00	0.00
N	167	165	162	159	156	144	132

**SEK/USD**	M1	M3	M6	M9	M12	M24	M36

RMSFE_M/RMSFE_B	0.87	0.63	0.40	0.32	0.28	0.18	0.15
MAFE_M/MAFE_B	0.92	0.76	0.59	0.52	0.48	0.37	0.34
DMW	0.01	0.00	0.00	0.00	0.00	0.00	0.00
CW	0.00	0.00	0.00	0.00	0.00	0.00	0.00
N	167	165	162	159	156	144	132

***Note:*** The dependent variable is the h-step forward difference of the given currency pair. The individual columns (Mx) denote the results for the x month ahead forecast. RMSFE_M/RMSFE_B denote the ratio of root mean squared forecast error of the DMA framework and Panel regression benchmark (fixed effects regression with real exchange rate as explanatory variable).MAFE_M/MAFE_B represents the ratio of the mean absolute forecast error between the two. DMW and CW indicate the p-values for the Diebold-Mariano and Clark-West test statistics, respectively. N refers to the number of estimated forecasts. DMA framework was used for the estimation. A rolling window approach was used for the estimation of the benchmark model.

## Data Availability

Data is not available to access in a data repository; however, it will be made available by the corresponding author upon request.

## Footnotes

2 We offer a brief description of these models in the next section.

3 [29] and [30] generally achieved better results with the unemployment rates/gaps as they found out that the use of unemployment rates/gaps increases out-of-sample exchange rate predictability for almost all of the specifications.

4 Alternatively, interest rates can also be used directly as explanatory variables.

5 Both models can be derived using uncovered interest parity relation, which includes the risk premium. For example, the foreign exchange (FX) risk premium can be interpreted as a convexity adjustment of the lognormal pricing kernels, specifically reflecting the difference between the convexity adjustments of the home and foreign countries [45], [46].

6 The parameter instability is based on a scapegoat story: some (fundamental) variable is given an ‘excessive’ weight during a certain period due to the belief that it influence the exchange rate. The exchange rate may change for reasons that have nothing to do with the observed (fundamental) variable. Facing incomplete and heterogeneous information, investors attach excessive weight to an observed variable. Over time, different variables can be taken as scapegoats so that the weights attributed to the variables change.

7 For clarification, we use the data regarding other explanatory (fundamental) variables for the countries of the respective exchange rates, which are listed above.

8 The motivation behind the inclusion of this variable is based on the monetary policy rule. Thus, we indirectly forecast interest rate changes, which, based on uncovered interest rate parity, cause movements in the exchange rate. Also, the implicit assumption is that we assume a constant natural rate of unemployment or that the use of time-varying coefficients also implicitly accounts for changes in the natural rate of unemployment. [30] use the non-accelerating inflation rates of unemployment (NAIRU) from the OECD Economic Outlook and unemployment rates to construct the unemployment gaps. On the other hand, [29] simply use (forecasted) unemployment rates. The authors argue that given the use of rolling regressions where the constant, as well as the coefficients, change each period, using the real-time unemployment rate is similar to using the real-time unemployment gap.

9 From an accounting perspective, these loans serve as asset counterparts to a portion of monetary aggregates. If we were to use total loans or broad monetary aggregates such as M2 or M3, which closely align with total loans, we would also incorporate items like mortgages. Mortgages, which have an average maturity exceeding 20 years in developed countries and account for approximately half of total credit, would introduce substantial persistence into the credit time series due to their long-term nature, effectively creating autocorrelation over two decades. An alternative approach would be to use only new (corporate) loans; however, such time series are not available for all countries.

10 When monthly data were unavailable, we employed piecewise constant interpolation to convert the series to monthly values. This approach ensures that unpublished data are not used in the forecasting exercise. For New Zealand, the monthly unemployment rate was constructed by using the registered unemployment figures, which are published monthly, alongside quarterly data on the labour force population to derive the rate. In the case of Japan, a time series of loans to all corporations was used as a proxy for money. This is because data on loans to non-financial corporations are not available.

11 Note that the Swiss National Bank implemented a one-sided exchange rate target zone with respect to the euro from September 2011 to January 2015. This policy may influence the results obtained and should be taken into consideration when interpreting the findings.

12 We were mainly limited by the inclusion of the EUR/USD currency pair as one of the most active currencies and also, in some cases, the availability of credit data.

13 To simplify the interpretation of the text, we often describe the results in terms of short, medium, and long-run periods. We generally consider a period of up to and including 6 months as a short-run period. A period from 7 to 12 months is considered a medium-run period, and more than one year is considered a long-run period.

14 Therefore, equation (8) and (9) estimated separately for each currency. Also, in this part, for better readability, we omit the subscript *k*, which indicates the different models regarding every currency pair *i*. This is because the DMA approach uses many different model specifications.

15 Since a distinct model is estimated for each forecast horizon and each currency, the lead length *h* varies according to the selected forecast horizon.

16 However, we abstract from the fact that if we assume rational expectations, the theoretical models described above generally imply forward-looking solutions. The results also depend on the rate of adjustment, i.e., if we assume that all adjustments are immediate or if there exists some smoothing. For example, interest rate smoothing, sticky prices, etc.

17 Therefore, the signs regarding these coefficients are generally unclear.

18 Most studies [17], [39], [40] employ a single-equation error correction model (ECM) striped of short-term adjustment terms, as this approach facilitates exchange rate forecasting using only the past values of other variables.

19 For ease of exposition, we restrict our attention only to the relevant Kalman filter formulas.

20 The results were generally robust to different specifications.

21 A related point is that some of the variables considered are non-stationary in levels. This is not a problem when considering the usual error-correction model specification but may be problematic because the cointegrating relationships are not present in all combinations in the DMA framework. For this reason, in the empirical part, we also focus on the stationarity of residuals.

22 We employ HAC estimator to calculate the variance.

23 Each section of the table follows the same structure.

24 The obtained out-of-sample forecasts were transformed back to levels to provide further intuition on results.

25 The implicit argument here is that both countries have the same inflation target. At the same time, due to the interconnectedness, inflationary pressures spill over between respective countries, and the central banks react similarly.

26 Posterior inclusion probabilities for individual variables are based on posterior model probabilities that contain the given variable.

27 However, the inclusion probability of fundamental variables is generally slightly increasing with the time horizon.

28 From a practical point of view, we posit that corporate deposits represent a key component of money influencing the nominal exchange rate, as they are most likely to be involved in international transactions. This focus allows us to filter out the ‘noise’ often present in broader monetary aggregates.

29 We again generally expect the coefficients $un_{US}$ and $ir_{US}$ to be positive and the coefficients $un_{domestic}$ and $ir_{domestic}$ to be negative, following uncovered interest rate parity and monetary policy rules. However, the issue regarding the correct signs is more complicated, as an increase in the interest rate should cause an immediate appreciation of the dollar, followed by forecasted (and actual) depreciation.

30 US unemployment can serve as a proxy for global economic conditions. As such, it may be a significant predictor of the exchange rate not only indirectly through the uncovered interest rate parity but also directly, as it serves as a proxy for global risk aversion and phenomena such as flight to quality.

31 We used all possible combinations of explanatory variables. There are $2^{K}-1$ possible models to construct, where *K* denotes all possible explanatory variables (including constant term).

32 We also experimented with the recursive (expanding) window approach, but the results with the recursive were significantly worse than with the rolling window. In short, the rolling window approach provides better predictions relative to the recursive window regarding RMSFE and MAFE ratios.

33 However, the use of rolling window estimation in practical terms enables time-varying coefficients.

34 Also, CW test statistics confirm these results.

35 Due to the article's length, the results are not shown in a separate table. Nevertheless, they (RMSFE and MAFE ratios) can be obtained using results from Table 1 and Table 4.

36 We, however, remind the readers that in this specification, the coefficients regarding price levels are fixed and are the same for every forecasting horizon.
